# Photosynthetic performance and photosynthesis-related gene expression coordinated in a shade-tolerant species *Panax notoginseng* under nitrogen regimes

**DOI:** 10.1186/s12870-020-02434-z

**Published:** 2020-06-28

**Authors:** Jin-Yan Zhang, Zhu Cun, Jun-Wen Chen

**Affiliations:** 1 College of Agronomy & Biotechnology, Yunnan Agricultural University, Kunming, 650201 China; 2 Key Laboratory of Medical Plant Biology of Yunnan Province, Yunnan Agricultural University, Kunming, 650201 China; 3 National & Local Joint Engineering Research Center on Germplasm Innovation & Utilization of Chinese Medicinal Materials in Southwestern China, Yunnan Agricultural University, Kunming, 650201 China

**Keywords:** Photosynthesis, Rubisco, Chloroplast, Non-photochemical quenching, Nitrogen, *Panax notoginseng*

## Abstract

**Background:**

Nitrogen (N) is an essential component of photosynthetic apparatus. However, the mechanism that photosynthetic capacity is suppressed by N is not completely understood. Photosynthetic capacity and photosynthesis-related genes were comparatively analyzed in a shade-tolerant species *Panax notoginseng* grown under the levels of low N (LN), moderate N (MN) and high N (HN).

**Results:**

Photosynthetic assimilation was significantly suppressed in the LN- and HN-grown plants. Compared with the MN-grown plants, the HN-grown plants showed thicker anatomic structure and larger chloroplast accompanied with decreased ratio of mesophyll conductance (g_m_) to Rubisco content (g_m_/Rubisco) and lower Rubisco activity. Meanwhile, LN-grown plants displayed smaller chloroplast and accordingly lower internal conductance (g_i_). LN- and HN-grown individuals allocated less N to light-harvesting system (N_L_) and carboxylation system (N_C_), respectively. N surplus negatively affected the expression of genes in Car biosynthesis (*GGPS*, *DXR*, *PSY*, *IPI* and *DXS*). The LN individuals outperformed others with respect to non-photochemical quenching. The expression of genes (*FBA, PGK, RAF2, GAPC, CAB, PsbA* and *PsbH*) encoding enzymes of Calvin cycle and structural protein of light reaction were obviously repressed in the LN individuals, accompanying with a reduction in Rubisco content and activity. Correspondingly, the expression of genes encoding *RAF2*, *RPI4*, *CAB* and *PetE* were repressed in the HN-grown plants.

**Conclusions:**

LN-induced depression of photosynthetic capacity might be caused by the deceleration on Calvin cycle and light reaction of photosynthesis, and HN-induced depression of ones might derive from an increase in the form of inactivated Rubisco.

## Background

Nitrogen (N) is a major limiting factor in natural ecosystems and in most agricultural systems [1, 2]. N is regarded as a necessary component of numerous biomolecules, such as DNA, RNA, proteins, chlorophyll (Chl) and cell envelope [3, 4]. N shortage results in enormous changes in plant morphology and even destroys the balance of biological process, including N metabolism and photosynthesis [5, 6]. N-deficient crops show the premature of leaves, and reduce leaf area expansion, plant height and ultimately yield of their own [5–9]. On the other hand, excessive N supply makes leaves dark green and stems frail and immature, and consequently cause an imbalance between the vegetative and reproductive growth [10–12]; For example, excessive N supply considerably reduces the biomass of cucumber (*Cucumis sativus*) [13] and of tomato (*Lycopersicon esculentum*) [14] . However, N surplus in plants receives relatively little attentions in comparison with N deficiency over the past decades.

It has been commonly accepted that photosynthesis is highly influenced by leaf anatomy and chloroplast ultrastructure. HN-grown *Arabidopsis. thaliana* displays thicker upper epidermises, lower epidermises, spongy tissue and palisade tissue, and increased thickness of anatomic structure would not facilitate CO_2_ diffusion in the liquid phase of mesophyll cells [15]. N deficiency exhibits small chloroplast with lower internal conductance (g_i,_) [16], and a large chloroplast with well-developed grana under high-N application has been reported in summer maize [17]. Indeed, Photosynthesis-related components are strongly regulated by leaf N and photosynthetic capacity is closely related to N content since more than 50% of total leaf N is allocated to photosynthetic machinery and proteins of Calvin cycle represent the majority of leaf N [18–21]. In leaves of developing maize (*Zea mays*), N deficiency results in an obvious decrease in photosynthesis with an reduction in activities of phosphor enolpyruvate carboxylase (PEPC), pyruvate orthophosphate di-kinase (PPDK) and ribulose 1, 5-bisphosphate carboxylase (Rubisco) [17, 22–24]. A reduction in content of Rubisco and in effective and maximum quantum yield of photosystem II (Δ*F*_v_/*F*_m_ & *F*_v_/*F*_m_) has been recorded in *A. thaliana* and *Oryza sative* grown under N deficiency condition [25, 26]. Likewise, Rubisco carboxylase activity considerably declines in spinach (*Spinacia oleracea*) and cassava (*Manihot esculenta*) due to N deficiency [27, 28]. On the other hand, negative responses of photosynthetic capacity to excess N, including decreased Rubisco activity, lower N allocation to light-harvesting system (N_L_) and lower photosynthetic efficiency, have been observed in field-grown wheat [29], rice [30] and cotton [31]. In general, the relationship between N levels and photosynthesis is nonlinear in a sufficiently broad range of leaf N content [32, 33]. However, relatively little is known about its molecular mechanism on the nonlinear relationship between leaf N and photosynthesis.

N-suboptimal plants would suffer from greater excess of light energy, and this could produce excessive reactive oxygen species (ROS) [34]. Plants has employed a series of photoprotective mechanism to survive long periods of no-optimal N regimes. Non-photochemical quenching (NPQ) of excess light energy within the light-harvesting antennae of PSII (LHCII) are believed as an effective photoprotective mechanisms, as observed in *A. thaliana* plants subjected to low nitrogen (LN) [26, 35, 36] and in *Coffea Arabica* [37] and *Lavandula angustifolia* [38]. Excess N supply in benthic diatom (*Entomoneis paludosa*) [39] have revealed an up-regulation of xanthophyll cycle, which reduce the efficiency of PSII photochemistry and enhance NPQ. In addition, several studies have highlighted a positive effect of glycolytic pathway and pentose phosphate pathway (PPP) on energy and carbon balance in N-stressed plants [40, 41]. Unexpectedly, molecular mechanisms of photoprotection are not completely clear in the N-stressed non-model species, especially in a shade-tolerant plant.

Comparative transcriptomes have revealed that unigenes expression of Chl biosynthesis, Calvin cycle and ribosomal proteins were decreased in *Scenedesmus acuminatus* under high N (HN) supply [42]. Up-regulated gene transcripts are predominantly matched in kinds of amino acid metabolism, transport and stress, whereas repressed transcripts are overrepresented in categories of hormone metabolism and redox control in roots of *A. thaliana* under N deficiency [43]. During periods of N-limitation, gata transcription factor (*GNC*), a gene regulating carbon (C) and N metabolism, operates to support *A. thaliana* survival by elevating Chl biosynthesis [44]. Genes encoding enzymes for C skeleton production are down-regulated in spinach plants under N-starvation, and plants also significantly show low contents of amino acid and high levels of glucose and consequently decelerate growth [45]. Light reaction center of photosynthesis by extrinsic proteins labled as *PsbO*, *PsbP*, *PsbQ*, *PsbR*, *PsbU* and *PsbV* are suppressed in Synechocystis under N stress [46]. *PsbS* protein is activated by the acidity of thylakoid lumen in *A. thaliana* plants under N-stressed condition [47]. In addition, the expression of *NR* and *GOGAT* was dramatically up-regulated in the cucumber exposed to HN [48]. Surprisingly, relatively less investigation has been conducted to elucidate the correlation of photosynthesis-related genes expression with photosynthetic performance in the context of N.

*Panax notoginseng* (Burkill) F. H. Chen (Sanqi in Chinese) is a typically shade-tolerant species from the family of Araliaceae [49–51], In our previous researches, *P. notoginseng* is believed to be highly sensitive to high light, and 10% of full sunlight is suitable for its growth [50, 52]*.* Besides, the development and growth of *P. notoginseng* is highly sensitive to high N [53–55]. HN application considerably enhance rust cracking, root decay and mortality rate of *P. notoginseng* [56]. Indeed, significant decreases in root, stem and leaf biomass have been observed in *P. notoginseng* grown under LN, along with narrow and yellow leaves [57, 58]. However, these previous studies have mainly focused on effects of N input on agronomic traits, yield, and plant growth. Nowadays, the molecular mechanism of the sensitivity of *P. notoginseng* to N is still unclear.

Different N levels were applied to *P. notoginseng*, and photosynthetic capacity, photoprotection and photosynthetic pigments were comparatively analyzed in the plants grown under low N (LN), moderate N (MN) and high N (HN). Meanwhile, a comprehensive transcriptome was conducted to elucidate the expression of photosynthesis-related gene. The objective of our study was to elucidate the photosynthetic performance and the expression of photosynthesis-related genes in the typically shade-tolerant and N-sensitive plant *P. notoginseng* under different levels of N, and it was anticipated that photosynthetic performance might be coordinated with the expression of photosynthesis-related genes.

## Results

### Effect of N regimes on plant growth and leaf gas change

**Fig. 1:**
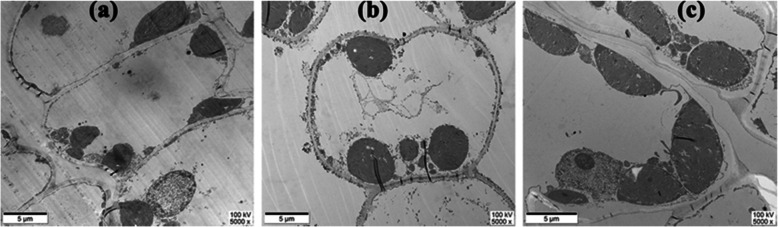
Electron micrograph of chloroplast with low(**a**), moderate(**b**) and high**(c)** nitrogen level were taken at 5000, 5000 and 5000 times, respectively

HN-grown leaves were pretty dark-green, and LN-grown leaves were significantly smaller and yellowish (Additional file 1: Figure S1a). HN-grown plants possessed a low survival rate (Additional file 1: Figure S1b). LN-grown leaves were dramatically reduced in the thickness of upper epidermis, lower epidermis, spongy tissue and palisade tissue, and biomass of leaf was significantly reduced in LN and HN treatments (Table 1). On the other hand, LN significantly decreased the size of chloroplasts accompanied with a reduction in chloroplast exposed to intercellular air space per unit leaf area (S_c_), and correspondlingly an increase in the size of chloroplasts was observed in *P. notoginseng* under excessive N supply (Fig. 1 c; Table 2). The LN plants and HN plants showed 52.7 and 96.8% lower liquid phase (g_lip_) than the MN plants, respectively. g_lip_ can be expressed as g_lip_ = C_lip_ × S_C_, therefore, conductance per unit of exposed chloroplast surface area (C_lip_) is one of a determinant of g_lip_. C_lip_ were reduced in HN-grown plants as compared to the MN-grown individuals. Internal CO_2_ condutance (g_i_) is mainly determined by g_lip_, and LN-and HN-grown plants was decreased in g_i_ (Table 2)_._

**Table 1:** Effects of nitrogen regimes on the leaf morphology, anatomy and biomass in a shade-tolerant plant *Panax notoginseng*

Variables		Nitrogen level	
	LN	MN	HN
Upper epidermis (μm)	11.209 ± 0.024 c	17.694 ± 1.927 a	14.738 ± 0.269 b
Lower epidermis (μm)	10.590 ± 1.027 c	13.177 ± 2.186 a	11.420 ± 0.918 b
Spongy tissue (μm)	42.551 ± 2.194 c	70.378 ± 0.182 a	56.518 ± 0.189 b
Palisade tissue (μm)	17.069 ± 1.283 c	32.867 ± 0.173 a	24.490 ± 1.825 b
Palisade/spongy	0.401 ± 0.002	0.467 ± 0.016	0.433 ± 0.016
Leaf length (cm)	6.484 ± 1.980 c	7.515 ± 1.068 a	6.795 ± 1.238 b
Max width (cm)	2.777 ± 0.698 c	3.114 ± 0.621 ab	3.273 ± 0.519 a
Leaf length/max width	2.341 ± 1.339	2.413 ± 0.8445	2.076 ± 0.879
Leaf dry weight (g plant^− 1^)	0.413 ± 0.040 c	0.545 ± 0.025 a	0.496 ± 0.064 b
Total dry weight (g plant^− 1^)	1.068 ± 0.294 c	1.649 ± 0.181 a	1.524 ± 0.088 b

Values are means ± SD. (*n* = 7). Different letters among nitrogen regimes indicate significant difference (*P* ≤ 0.05)

**Table 2:** Effects of N regimes on leaf photosynthesis in *Panax notoginseng*

Variables	LN	MN	HN
g_s_ (mol CO_2_ m^− 2^·s^− 1^)	0.05 ± 0.02 a	0.03 ± 0.02 ab	0.03 ± 0.02 ab
g_m_ (mol CO_2_ m^− 2^·s^− 1^)	0.09 ± 0.01 c	0.26 ± 0.04 b	0.36 ± 0.02 a
R_d_ (μmol CO_2_ m^− 2^·s^− 1^)	1.0 ± 0.04 a	0.52 ± 0.02 b	0.57 ± 0.03 b
g_lip_ (mol CO_2_ m^− 2^·s^− 1^)	2.61 ± 0.08 b	5.52 ± 0.03 a	0.183 ± 0.09 c
*C*_c_ (μmol CO_2_ m^− 2^·s^− 1^)	199.53 ± 8.27 b	265.45 ± 7.31ab	291.58 ± 9.15 a
S (mol mol^− 1^)	844.15 ± 7.56 b	1057.25 ± 5.41 a	860.16 ± 3.89 b
S*(mol mol^− 1^)	739.39 ± 95.61b	983.75 ± 67.32 a	753.41 ± 90.34 b
Rubisco activity (nmol/min/g)	0.643 ± 0.24 c	40.51 ± 5.39 a	22.51 ± 4.89 b
Rubisco content (μg/g^−1^)	6.931 ± 0.36 c	10.057 ± 0.67 b	70.494 ± 0.32 a
S_c_(m^2^ m^− 2^)	8.42 ± 1.25 b	12.01 ± 1.65 a	13.15 ± 0.56 a
C_lip_ (mol CO_2_ m^− 2^·s^− 1^)	0.31 ± 0.06 ab	0.46 ± 0.02 a	0.02 ± 0.01 b
g_i_ (mol CO_2_ m^− 2^·s^− 1^)	0.13 ± 0.01 b	0.35 ± 0.04 a	0.12 ± 0.05 b
g_m_/Rubisco content	12.99 ± 1.23 b	25.81 ± 1.90 a	5.12 ± 0.78 c

Values are means ± SD. (*n* = 7). Different letters among nitrogen regimes indicate significant difference (*P* ≤ 0.05). g_s_: stomatal conductance; g_m_: mesophyll conductance; R_d_: dark respiration rate; g_lip_: liquid phase; *C*_c_: chloroplastic CO_2_ concentration; S: the specificity factor of Rubisco for O_2_ and CO_2_; S*: apparent Rubisco specificity; S_c_: chloroplast exposed to intercellular air space per unit leaf area; C_lip_: conductance per unit of exposed chloroplast surface area; g_i_: internal CO2 condutance

### N-induced changes in photosynthetic capacity

**Fig. 2:**
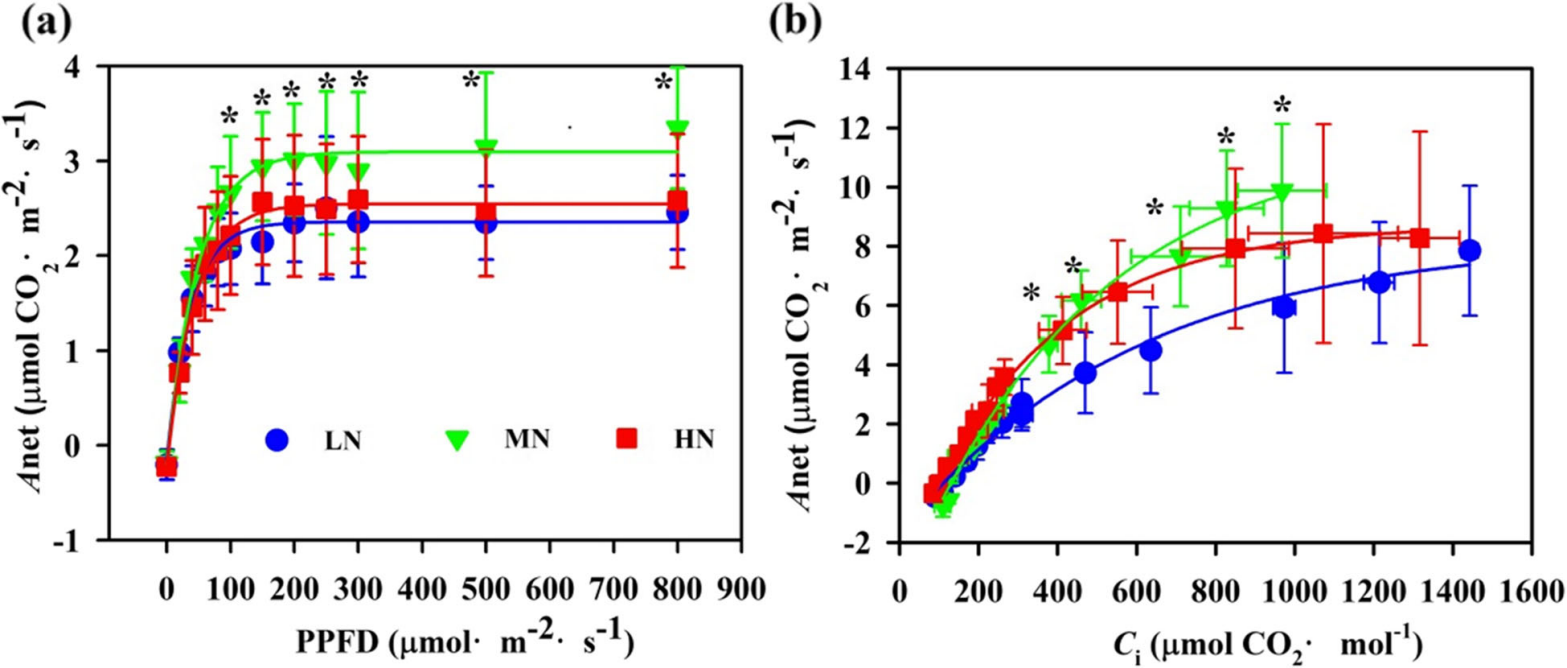
**a** Response of net photosynthetic rate (*A*_net_) to photosynthetic photon flux density (PPFD) in *Panax notoginseng* grown under low nitrogen (LN), moderate nitrogen (MN), high nitrogen (HN). **b** The change of net photosynthetic assimilation (*A*_net_) with intercellular CO_2_ concentration (*C*_i_) in *Panax notoginseng* grown under different nitrogen levels. Values for each point were means ± SD (*n* = 7). Significant differences are indicated by asterisks (ANOVA; *P* values ≤0.05)

**Fig. 3:**
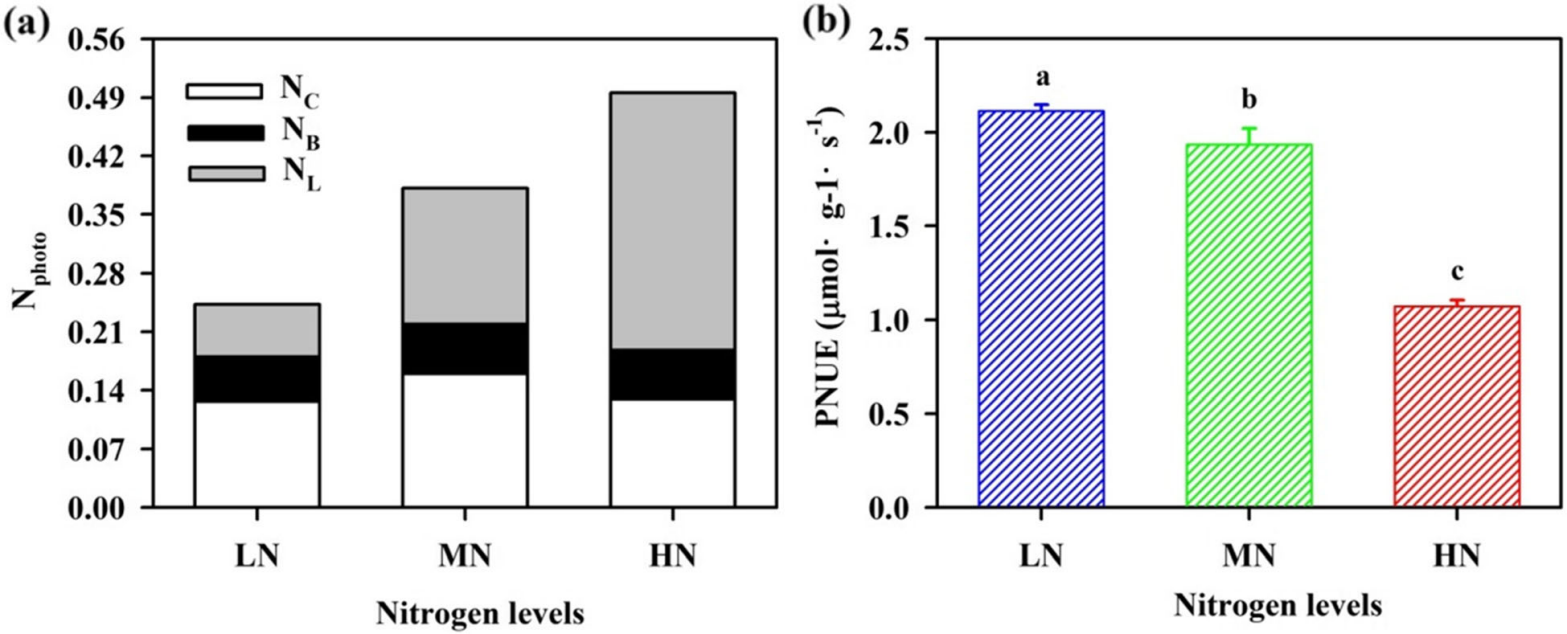
Effects of different nitrogen levels on nitrogen distribution (*n =* 7) and photosynthetic nitrogen use efficiency (*n* = 7) in *Panax notoginseng* leaves. N_photo_: Photosynthetic apparatus; N_C_: Carboxylation system; N_B_: Bioenergetics; N_L_: Light harvesting system; PNUE: Photosynthetic nitrogen use efficiency. Data are mean with bars depicting standard deviation (± SD). Significant differences are indicated by letters (ANOVA; *P* values ≤0.05)

The leaf exhibited a significant difference in a response of net photosynthetic assimilation (*A*_net_) to incident photosynthetic photon flux density (PPFD) and to internal leaf CO_2_ concentrations (*C*_i_) within N regimes (Fig. 2). The maximum net photosynthetic assimilation (*A*_max_), CO_2_ response curves and carboxylation efficiency (CE), maximum electron transfer rate (*J*_max_)and maximum carboxylation efficiency (*V*_cmax)_ were highest in MN- grown plants; however, these variables did not show apparent differences between LN and HN individuals except for *A*_max_ (Table 3). N allocation to the photosynthetic system (N_photo_) is the sum of N allocation to the carboxylation system (N_C_), the bioenergetics component (N_B_) and the light-harvesting system (N_L_). N content per unit leaf area (SLN) was increased significantly with the increase in N application (Table 3). HN treatment caused a significant increase in N_L_, whereas there is a significant reduction in N_C_ in HN-grown plants (Fig. 3 a). Most importantly, photosynthetic N use efficiency (PNUE) was significantly decreased from 45.2 to 20.3% with an increase in N supply (Fig. 3 b). These results support that high N_photo_ did not trigger an increase in PNUE.

**Table 3:** Steady-state photosynthetic-related traits in *Panax notoginseng* under different levels of nitrogen

Variables	Nitrogen level		
	LN	MN	HN
*A*_max_ (μmol·m^− 2^·s^− 1^)	2.378 ± 0.261c	3.437 ± 0.241a	2.600 ± 0.165 b
CE (mol·mol^−1^)	0.017 ± 0.002 ab	0.022 ± 0.003 a	0.018 ± 0.005 ab
Γ*(μmol·mol^− 1^)	124.399 ± 8.014 a	99.259 ± 10.957 b	122.121 ± 21.084 ab
*J*_max_ (μmol·mol^− 1^)	66.558 ± 6.123 b	74.518 ± 15.599 a	63.334 ± 23.251b
*V*_cmax_ (μmol·mol^− 1^)	16.480 ± 1.821b	20.771 ± 2.939 a	16.830 ± 5.058 b
*J*_max_/*V*_cmax_	4.059 ± 0.127 ab	3.527 ± 0.337 b	4.329 ± 0.106 a
SLN (g m^− 2^)	0.890 ± 0.130 c	1.245 ± 0.006 b	2.178 ± 0.348 a

Values are means ± SD. (*n* = 7). Different letter among nitrogen treatments represents a significant level (*P* ≤ 0.05). *A*_max_: maximum photosynthetic assimilation at the saturating light; CE: carboxylation efficiency; Γ*: carbon dioxide compensation point; *J*_max_: maximum electron transfer rate; *V*_cmax_: maximum carboxylation efficiency; SLN: nitrogen content per unit leaf area

SLN and Rubisco content were greater when plants were exposed to high N as compared with ones to moderate and low N (Additional file 2: Figure S2; Tables 2, 3). MN treatment exhibited 44.4–98.4% more Rubisco activity than two other treatments (Table 2). There were no significant differences in stomatal conductance (g_s_) within treatments, but g_m_ were higher in HN-grown individuals (Table 2). HN-grown plants had a decreased ratio of g_m_/Rubisco content and a lower Rubisco specific activity than two other treatments (Table 2).

### Photosynthetic electronic transport

**Fig. 4:**
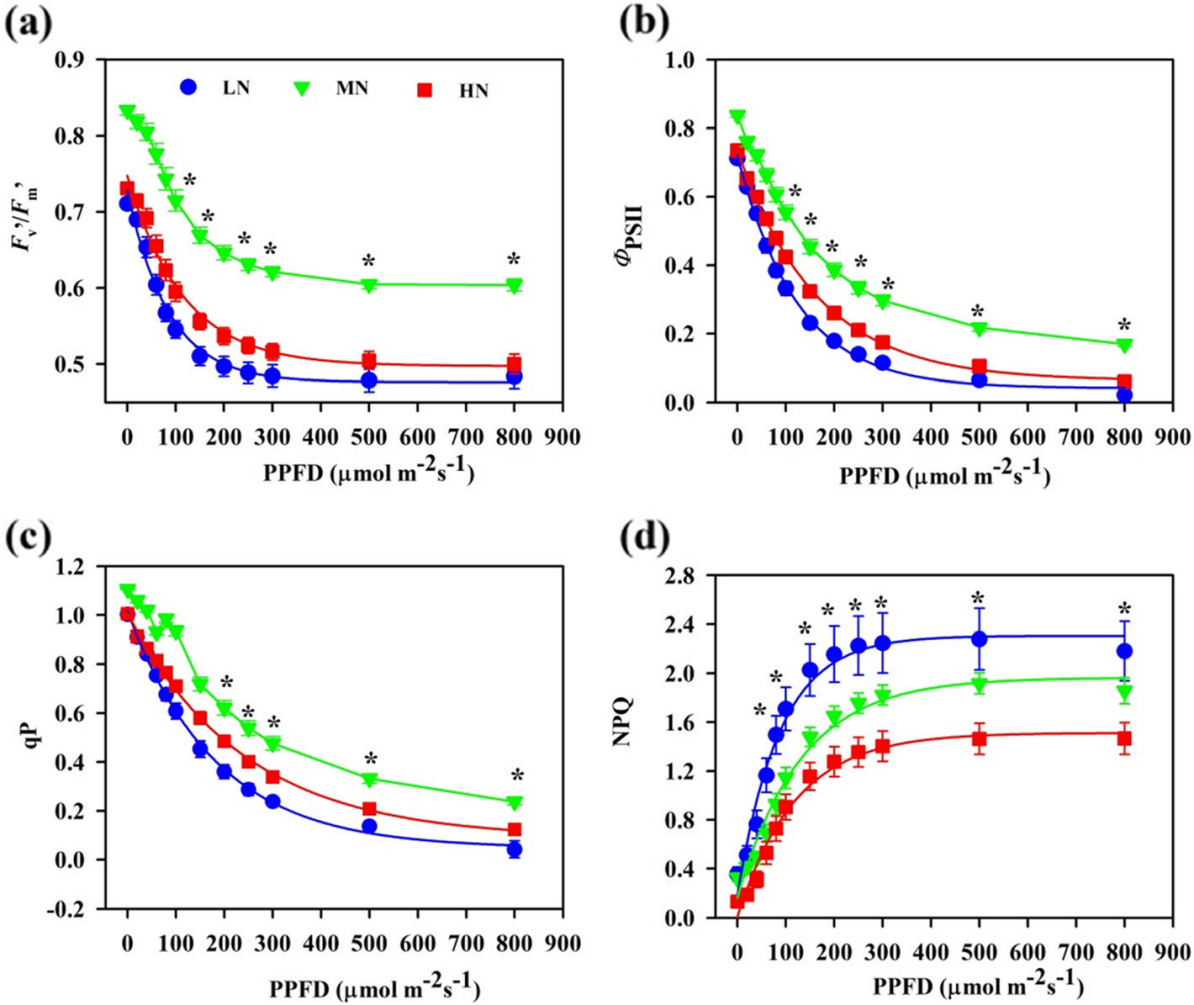
Responses of PSII maximum quantum efficiency (*F*_v_`/*F*_m_`, **a**), PSII photochemical quantum yield (*Φ*_PSII_, **b**), photochemical quenching (qP, **c**), non-photochemical quenching (NPQ, **d**) to photosynthetic photon flux density (PPFD) in *Panax notoginseng* grown under different levels of nitrogen. Values for each point were means ± SD (*n* = 7). Significant differences are indicated by asterisks (ANOVA; *P* values ≤0.05)

The responses of photosynthetic electronic transport to continuous steady-state light were markedly different among N regimes (Additional file 3: Figure S3; Fig. 4). In the light response curves, the minimum values of PSII maximum quantum efficiency(*F*_v_`/*F*_m_`), PSII photochemical quantum yield (*Φ*_PSII_), photochemical quenching(qP) as well as PSII total electron transport rate (*J*_T_), rate of electron transport for oxidation reaction (*J*_O_), carboxylation reaction (*J*_C_) and the maximum values of non-photochemical quenching (NPQ) were generally recorded in the LN individuals, the maximum value of *F*_v_`/*F*_m_`, *Φ*_PSII_, qP as well as *J*_T_, *J*_O_, *J*_C_ were obtained in the MN ones (Additional file 3: Figure S3; Fig. 4).

### Changes in photosynthetic-related pigments

The amounts of β-carotene (β- Cars) and the ratio of (V + A + Z)/Chl were enhanced in LN individuals, whereas total Chl decreased (Table 4). LN resulted in a decrease in neoxanthin (N) and lutein (L), and an increase in violaxanthin(V), antheraxanthin (A), and zeaxanthin(Z). Violaxanthin de-epoxide activity ((A + Z)/(V + A + Z)) was greatest in the LN ones (Table 4).

**Table 4:** Photosynthetic-related pigment in a shade-tolerant plant *Panax notoginseng* grown under different levels of nitrogen, means ± SD were given (*n* = 7)

Variables	Nitrogen Level		
	LN	MN	HN
N (μg·cm^−2^)	0.362 ± 0.129 c	0.865 ± 0.265 b	1.643 ± 0.332 a
V (μg·cm^− 2^)	0.913 ± 0.124 a	0.267 ± 0.195c	0.493 ± 0.458b
A (μg·cm^− 2^)	0.213 ± 0.019 a	0.043 ± 0.072c	0.153 ± 0.079b
L (μg·cm^− 2^)	1.284 ± 0.352 c	3.018 ± 0.970 b	5.852 ± 0.926a
Z (μg·cm^− 2^)	0.194 ± 0.023 a	0.032 ± 0.048 c	0.073 ± 0.201b
Chl(g·cm^− 2^)	12.270 ± 1.783 c	31.618 ± 2.356 b	60.101 ± 2.455 a
β-Car(g·cm^− 2^)	4.08 ± 2.14 a	1.59 ± 0.69 c	2.95 ± 0.69 b
V + A + Z (g·cm^− 2^)	1.314 ± 0.023 a	0.332 ± 0.035 c	0.712 ± 0.043 b
(A + Z)/(V + A + Z)	0.309 ± 0.015 ab	0.226 ± 0.017 b	0.317 ± 0.037 a
(V + A + Z)/Chl	0.107 ± 0.018 a	0.011 ± 0.027 b	0.012 ± 0.028 b

Different letter among nitrogen treatments represents a significant level (*P* ≤ 0.05). *V* violaxanthin; *A* antheraxanthin; *Z* Zeaxanthin; *L* Lutein; *N* Neoxanthin; β-Car: β-Carotene

### Gene expression identification

**Fig. 5:**
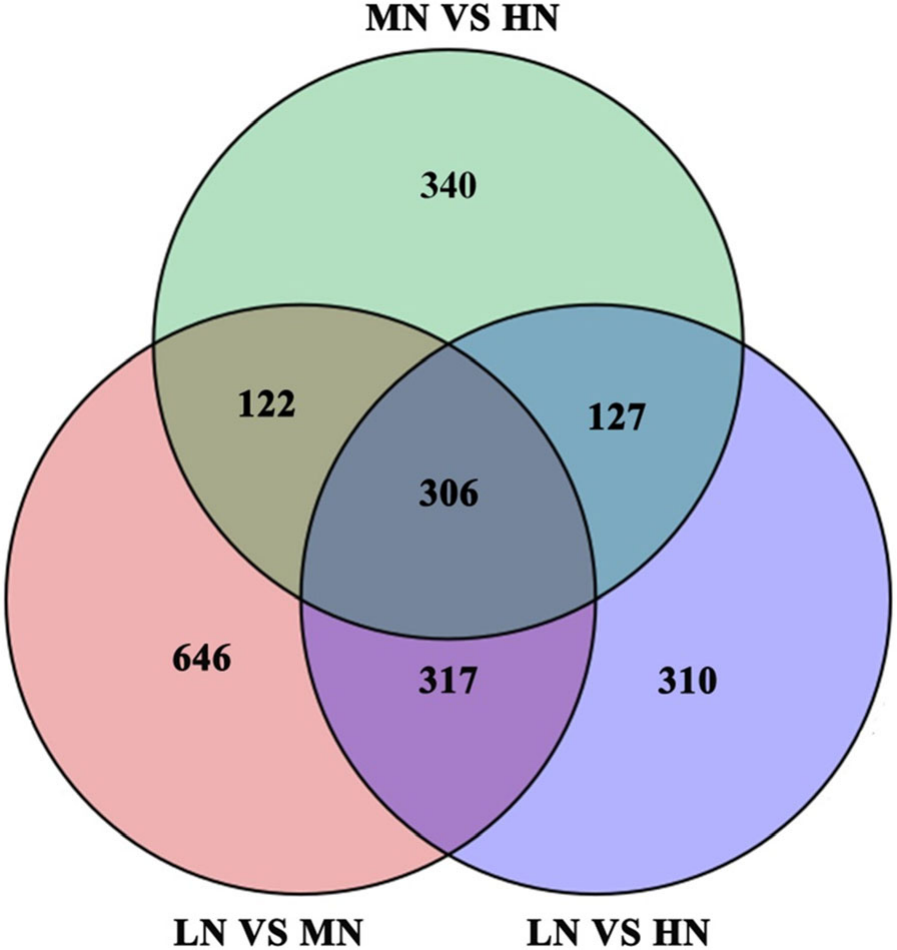
Venn diagrams of differentially expressed genes (DEGs) in response to varied nitrogen level

**Fig. 6:**
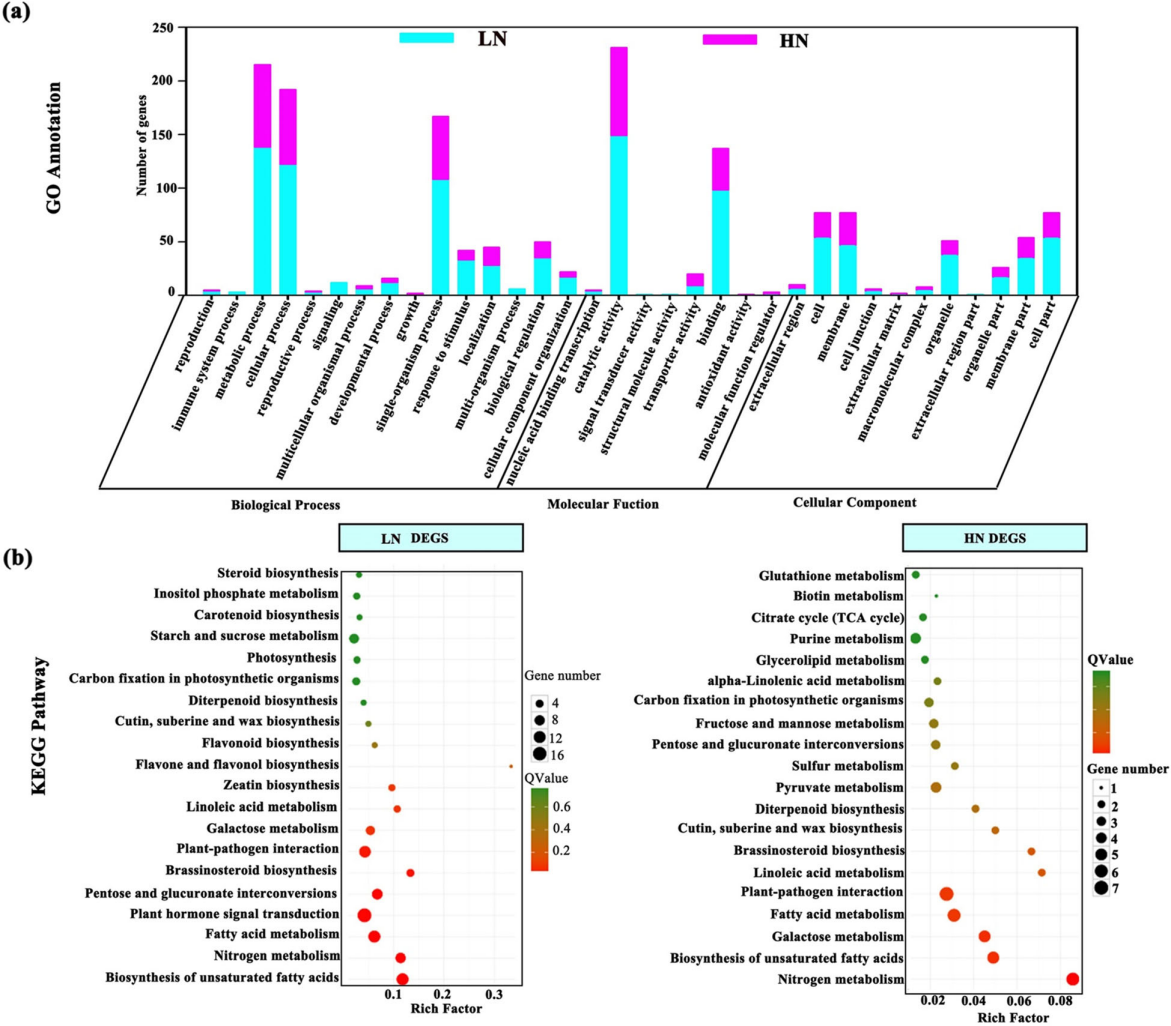
Functional annotation and enrichment analysis of differentially expressed genes (DEGs) responsive to low nitrogen and high nitrogen. **a** Gene annotation of DEGs. **b** KEGG enrichment analysis for DEGs

Compared to the MN individuals, 1391 and 895 genes were classified as differentially expressed genes (DEGs) in the LN and HN groups. Whereas, there were 428 DEGs in both LN- and HN- treatments (Fig. 5). In the LN group, 467 DEGs were up-regulated, and 924 DEGs were down-regulated. Two hundred ninety-four genes were up-regulated and 601 genes were suppressed in HN individuals (Additional file 4: Figure S4). Moreover, 963 and 467 DEGs were typically detected in LN, HN groups. Two DEG sets were subjected to 34 Gene ontology (GO) classes (Fig. 6). Under the classification of molecular function, “catalytic activity” were largely represented, followed by “binding” (Fig. 6 a). The GO enrichment was further analyzed to identify specific GO enrichment terms among DEG sets. Based on Kyoto Encyclopedia of Genes and Genomes (KEGG) pathway enrichment analysis, the DEGs in LN ones were categorized into photosynthesis, carbon fixation, N metabolic, plant hormone signal transduction, starch and sucrose metabolism and galactose metabolism, the DEGs in HN ones were significantly overrepresented in pathway of citrate cycle (TCA cycle), alpha-Linolenic acid metabolism, carbon fixation in photosynthetic organism, N metabolism and galactose metabolism (Fig. 6 b). In addition, the first 13 pathways widely related to the mechanism about photosynthesis and photo-protection were explored among KEGG enrich analysis of all annotated unigenes (Additional file 5: Table S1).

### Transcriptional changes

**Fig. 7:**
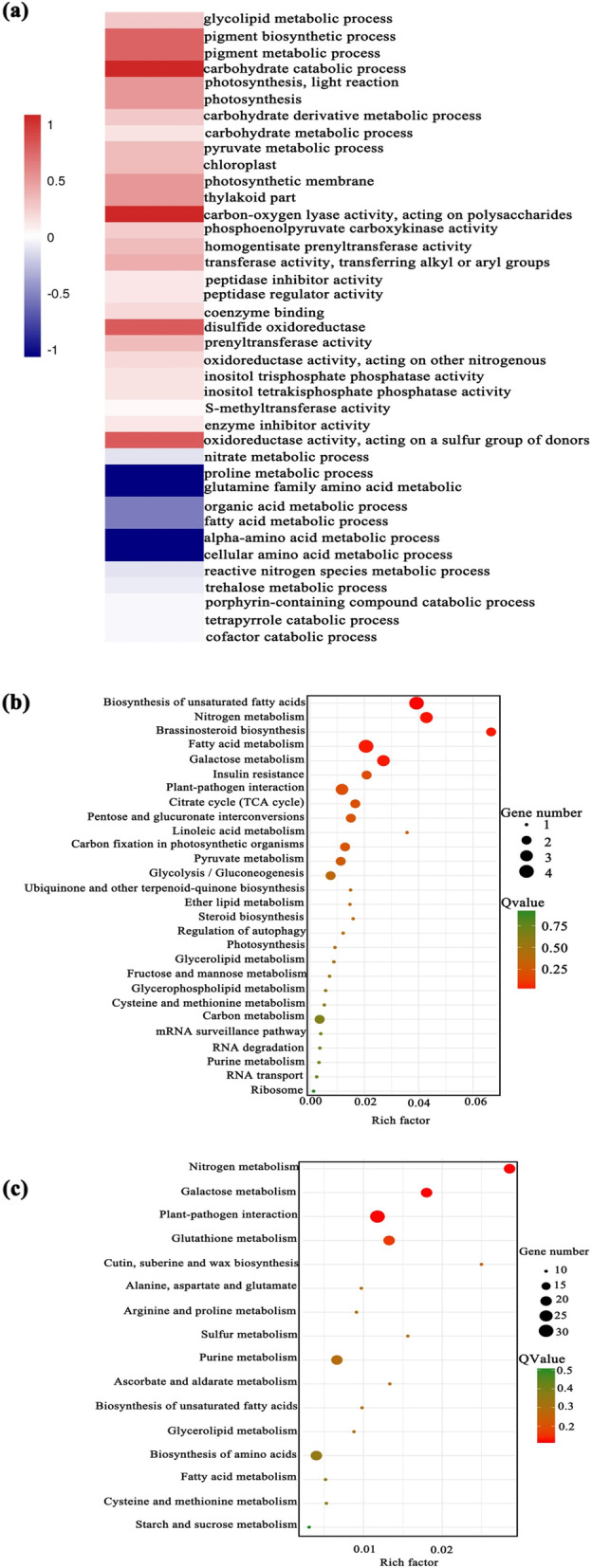
Functional annotation and enrichment analysis of common differentially expressed genes (DEGs) between moderate- (MN) vs. low- (LN) nitrogen and high-nitrogen (HN) vs. LN comparisons. **a** Heat clustering of common DEGs based on the expression profiles. Blue indicates lower expression, and red indicates higher expression. **b** KEGG pathway analysis of up-regulated DEGs. **(c)** KEGG pathway analysis of down-regulated DEGs

GO enrichment analysis was presented and elucidated in Fig. 7. Enriched GO terms of further induced genes between two pairwise comparisons (MN vs LN., MN vs HN.) embraced photosynthesis, pigment metabolic process, carbohydrate catabolic process, thylakoid and so on. Common DEGs with suppressed expression were significantly enriched in cellular amino acid catabolic process, alpha-amino acid catabolic process, proline metabolic process and glutamine family amino acid metabolic (Fig. 7). KEGG pathway analysis was further certified distinct functional enrichments in biological process among common DEGs (Fig. 7 b, c), revealing that these induced expression of common DEGs were richen in TCA cycle, photosynthesis, carbon fixation in photosynthetic organism, glycolysis/gluconeogenesis and carbon metabolism, and down-regulated DEGs were primarily related to N metabolism, glutathione metabolism, biosynthesis of amino acids and starch and sucrose metabolism. In addition, a large number of specific DEGs involved in diverse biological processes were detected in the MN vs. HN ones, and response patterns in the LN and HN level also exhibited differences.

### Genes expression related to Calvin cycle and light reaction

**Fig. 8:**
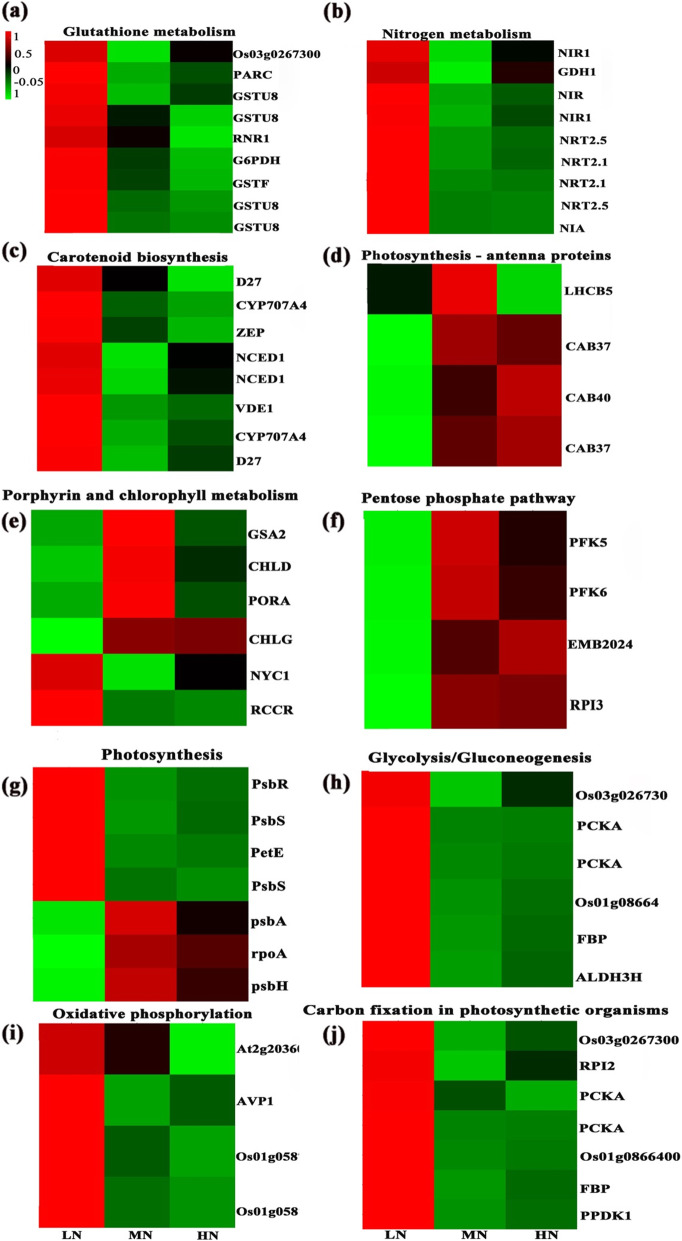
Expression profiles of differentially expressed genes (DEGs) that regulate photosynthesis and photoprotection under different nitrogen level

Interestingly, the expressions of the majority of genes encoding enzymes in Calvin cycle were down-regulated between LN and HN individuals, furthermore, the transcript levels of a substantial number of genes were reduced in LN ones (Additional file 6: Figure S5; Fig. 8). Expression of unigenes involved in photosystems II (e.g., *PsbA*, *PsbE*, *PsbF*, and *PsbH*) and photosystems I (e.g., *PsaN*) were down-regulated between LN and HN individuals (Additional file 7: Figure S6a; Fig. 8), while the unigenes involved in *PsbS* and *PetE* were up-regulated (Additional file 7: Figure S6b; Fig. 8).

### Photoprotection-related genes

Both zeaxanthin epoxidase (*ZEP*) and violaxanthinde-epoxidase (*VDE*) genes are positively induced by LN level (Additional file 8: Figure S7a). Glucose-6-phosphate dehydrogenase (*G6PDH*) and glutathione S-transferase (*GSTs*) involved in glucose metabolism was up-regulated in the LN individuals (Additional file 8: Figure S7b). Genes were found to be enriched in Chl degradation, and genes encoding chlorophyll b reductase (*NYC*) and red chlorophyll catabolite reductase (*RCCR*) were up-regulated in the LN groups (Additional file 8: Figure S7c). Under LN levels, transcript levels of genes involved in nitrate reduction were up-regulated (Additional file 8: Figure S7d). Expressions of genes encoding phytoene synthase (*PSY*), geranyl pyrophosphate synthase (*GGPS*), 1-deoxy-D-xylulose-5-phosphate reductoisomerase (*DXR*), isopentenyl pyrophosphate isomerase (*IPI*) and 1-deoxy-D-xylulose-5-phosphate synthase (*DXS*) were up-regulated in LN groups (Additional file 8: Figure S7e). In addition, a substantial number of pathway regulating photosynthesis and photoprotection were activated by N-induction, including glutathione metabolism, N metabolism, carotenoid biosynthesis, photosynthesis-antenna proteins (Fig. 8).

### Real-time quantitative PCR (RT-qPCR) of photosynthetic-related genes

In RT-qPCR analyses, melting curves of actin and 19 photosynthetic-related genes were clear and every curve all held a single and sharp peak (Additional file 9: Figure S8), indicating that the primer pairs could positively amplify specific products of 19 genes (Additional file 10: Table S2). The expressions of 19 genes were approximately similar to the results from RNA-Seq data in leaves (Additional file 11: Figure S9).

## Discussion

### N-driven changes in photosynthesis is in part explained by leaf anatomy and N allocation

Photosynthetic capacity is at least in part determined by leaf anatomy and chloroplast ultrastructure [59] and *A*_net_ is limited by the rate of CO_2_ diffusion from the atmosphere to the chloroplast [60]. C_lip_ has a close relationship with mesophyll cell thickness, and a thick tissue is always accompanied with a low C_lip_ [30]. Nevertheless, C_lip_ is positively related to the rate of CO_2_ diffusion from the atmosphere to the chloroplast [61]. N deficiency obviously reduce the size of chloroplasts and consequently lead to a low chloroplast surface area exposed to S_c_ [16]. Correspondingly, a large chloroplast has been documented in HN-grown rice, and the large chloroplasts would enhance chloroplastic CO_2_ concentration (C_c_) and g_m_ [62]. Thicker upper epidermis, lower epidermis, spongy tissue and palisade tissue (Table 1) would reduce liquid phase diffusion of CO_2_ in mesophyll cells, evidencing by lower C_lip_ in the HN individuals (Table 2), and this might partly explain the fact that a significant decrease in *A*_net_ was observed in the HN individuals. The increase in size of chloroplast and in g_m_ would contribute to the increase in *C*_c_ (Fig. 1 c; Table 2), as has also been observed in Tosens & Laanisto [63]. *C*_c_ positively reinforce photosynthetic capacity [64–66]. Nevertheless, HN supply resulted in a decline in *A*_net_ (Fig. 2), it has been thought that the increase in g_m_ is not enough to provide sufficient CO_2_ to activate the increased Rubisco, the imbalance between g_m_ and Rubisco content contributes to the reduction in *A*_net_ in the HN individuals (Table 2) as has also been observed in Yin & Struik [67]. LN-grown plants displayed small chloroplast and low S_c_ (Fig. 1; Table 2) and these characteristics suppress *g*_i_ and *A*_net_, as has been proposed by Onoda et al [68] .

30–40% of leaf N is allocated to photosynthetic carboxylation and photosynthetic efficiency is determined by the proportion of N allocated to N_C_ [69]. SLN is significantly increased, but *V*_cmax_, CE, Rubisco activity and N_C_ is obviously decreased, and consequently photosynthetic efficiency is suppressed in wheat and rice grown in excessive N environment [29, 30]. The LN plants exhibited lower SLN, Chl and N_L_ (Tables 3, 4; Fig. 3 a), this would only limit the synthesis of light-trapping chlorophyll a/ b-protein complexes and effectively prevent absorption of excessive light energy [70]. Correspondingly, lower N_C_ and activity of Rubisco were observed in the HN individuals (Fig. 3 a), this would suppress photosynthetic carboxylation and reduce photosynthetic efficiency.

### The dark and light reaction of photosynthesis under non-optimal N regimes

Rubisco is the key CO_2_-fixing enzyme in Calvin cycle. The depressed photosynthesis in the LN plants might be the consequence of low Rubisco catalytic capacity and CE [30, 71]. Rubisco activity and CE were decreased in the LN individuals with down-regulated expression of genes encoding Rubisco (Tables 2, 3; Additional file 6: Figure S5). N deficiency in maize led to the decline in photosynthetic capacity and Rubisco catalytic capacity [22]. According to classic theories [72], *V*_cmax_ positively reflects potential carboxylation capacity of Rubisco and regeneration rate of ribulose-bisphosphate (RuBP). *V*_cmax_ and Rubisco activity were considerably reduced in the HN individuals, whereas Rubisco content were increased (Tables 2, 3). On the other hand, the expressions of Rubisco-catalyzing genes (*CPN60A1*, *Os02g079470025* and *RAF2*) were down-regulated in the HN individuals (Tables 2, 3; Additional file 6: Figure S5). Overall, these results support the view that under HN supply, the majority of Rubisco function as N storage rather than as catalyzing enzyme [30], and the proportion of inactive Rubisco is greater in the HN leaves. However, high Rubisco activity is recorded in the HN-grown maize with high *A*_max_ [73]. The difference might be explained by the fact that *P. notoginseng* is a shade-tolerant C3 species and highly sensitive to excess N [54, 55], and maize is a high N- and sun-demanding C4 plant [74, 75]. In the atmospheric environment, Rubisco has a low affinity with CO_2_, resulting in inferior catalytic capacity [76]. The potential catalytic capacity of Rubisco in C3 plants is much lower than in C4 plants [77], suggesting that relatively less Rubisco operate on photosynthesis, and that a high proportion of Rubisco serves as a storage of N in HN-grown C3 plants.

The expression of genes encoding enzymes involved in regeneration of RUBP was reduced in the LN individuals, including phosphor ribulokinase (PRK), phosphor glycerate kinase (PGK), glyceraldehyde-3-phosphate dehydrogenase (GAPDH), triosephosphate isomerase (TPI), fructose-bisphosphate aldolase (FBA), transketolase (TKT) and sedoheptulose 1,7-bisphosphatase (SBP, Additional file 6: Figure S5), and correspondingly *A*_net_, *Φ*_PSII_, *F*_v_`/*F*_m_` and *J*_T_ were reduced. Likewise, N deficiency suppress photosynthetic capacity and inhibit the expression of genes involved in dark reaction of photosynthesis in barley (*Hordeum vulgare*) [78] and rice [79]. Our findings are in general agreement with several previous researches that down-regulated expressions of genes encoding enzymes involved in the regeneration of Rubisco is associated with abiotic stress [80, 81].

Photosynthetic assimilation and photosynthetic electronic transport was considerably decreased in the LN and HN individuals (Figs. 2, 4; Additional file 3: Figure S3), and it is believed to derive from an interruptive synthesis of photosynthetic proteins [82]. The expression of genes encoding structural proteins of photosystems, including *PsbA*, *PsbE*, *PsbF*, *PsbH* and *PsaN*, were decreased in the LN- and HN-grown individuals, while the expression of genes encoding subunits of the PsbS and PetE was increased as compared with the MN individuals (Additional file 7: Figure S6a). The expression of genes encoding structural proteins of photosystems are positively correlated with photosynthetic capacity [83, 84]. In addition, the down-regulation of genes involved in porphyrin and Chl metabolism and photosynthesis-antenna proteins might also in part explain the depressed photosynthetic capacity in the LN individuals (Fig. 8).

### Photoprotection in N deprivation

Lower *A*_max_, *V*_cmax_ and *J*_max_ were recorded in the LN and HN individuals when compared with the MN individuals (Table 3). No-optimal N application induces suppressions of photosynthesis as has been recorded in the sun-demanding species *Viciafaba* [85], *Lemna minor* [86], *Z. mays* [87] and *C. sativus* [48], and in the shade-tolerant species *Abies fabri* [88], *Brassica juncea* [89] and *polypodiopsida* [90]. On the other hand, *Φ*_PSII_ was lower in the LN individuals than in the HN individuals (Fig. 4 b), indicating that a greater proportion of light energy absorbed by PSII would have to be expended by non-photochemical process in the LN individuals. The consumption of electrons by non-photochemical process would facilitate the formation of trans-thylakoid ΔpH [86]. ΔpH is also a precondition for the activation of VAZ cycle and NPQ [91]. This indicates that NPQ and V cycle pool might be reinforced in the LN individuals as confirmed by the present study (Fig. 4 d; Table 4).

The degradation of Chl has been believed to be a photoprotective mechanism for plants to cope with stress and to prevent photodamage [92, 93]. Chl was considerably reduced in LN-grown plants (Table 4). Genes involved in Chl degradation were found to be enriched in LN-grown plants, and genes encoding *NYC* and *RCCR* were up-regulated (Additional file 8: Figure S7c). The results obtained in our study indicate that low Chl in LN-grown plants might be caused by up-regulation of genes involved in Chl degradation. Similarly, the expression of genes involved in Chl degradation are also elevated in a shade-tolerant plant *Neoregelia cruenta* when grown under N-limited condition, together with the obvious decrease in leaf Chl [40]. LN-grown Spinach showed low Chl content and the corresponding elevation in expression of genes involved Chl degradation [94, 95].

Car is not comprised of N atoms and their accumulation is beneficial for protecting photosystem from photodamage [42, 96]. The genes involved in Car biosynthesis (*GGPS*, *DXR*, *PSY*, *IPI* and *DXS*) were down-regulated in the HN individuals (Additional file 8: Figure S7e), as has also been observed by Vidhyavathi et al [97]. Correspondingly, the expression of genes involved in Car biosynthesis were up-regulated in *Haematococcus pluvialis* by a combination of light and N-deprivation [98]. Further, a greater xanthophyll pool size (V + A + Z) and higher de-epoxidation state ((A + Z)/(V + A + Z)) were observed in the LN individuals (Table 4), this suggests that LN cloud effectively improve the capacity of heat dissipation. The *PsbS* protein and violaxanthin de-epoxidase is believed to involve in regulating energy dissipation [93]. The latter catalyses the de-epoxidation of V to Z [99]. ΔpH-dependent quenching (qE) is activated by *PsbS* and the xanthophyll cycle [100]. The expression of genes encoding ZEP and VDE (key enzymes in xanthophyll cycle) were up-regulated in the LN individuals with the enhanced expression of *PsbS* genes (Additional file 8: Figure S7a), as has been observed in LN-induced maize where genes involved de-epoxidation state were up-regulated [34].

Nitrate assimilation is a process highly sensitive to N stresses [101]. Nitrite assimilation consumes six electrons from reduced ferredoxin [102, 103]. This reaction is a strong alternative sink for photosynthetic electron transport chain. The expressions of genes encoding nitrite reductase (*NIR*) and nitrate reductase (*NIA*) were increased in the LN-grown individuals (Additional file 8: Figure S7d). It has been reported that maize [74], *A. thaliana* [104] and apple [105] show high activity of NIR and NIA in leaves in presence of suboptimal N application. The previous investigations and the present study both strongly evidence that LN-induced enhancement in nitrate assimilation might mitigate the accumulation of excess energy.

## Conclusion

The non-optimal N supply significantly suppresses photosynthetic capacity in a typically shade-tolerant and N-sensitive species such as *P. notoginseng*. Thick leaf limits liquid phase diffusion of CO_2_ in mesophyll cells and accordingly reduces internal conductance. Moreover, large chloroplast with low N_c_ results in an imbalance between the increases in gm and in Rubisco content, consequently causing the decreased *A*_net_ in the HN individuals. In addition, the expression of genes involved in Calvin cycle, Chl biosynthesis and antenna proteins are obviously repressed in the LN individuals; correspondingly, the expression of genes (e.g. *RAF2*, *CAB* and *PetE*) involved in Calvin cycle and light reaction is also inhibited, however, photosynthetic capacity might be primarily inhibited by the inactivated Rubisco in the HN individuals. Overall, our results indicate that photosynthetic performance and photosynthesis-related genes expression is coordinated in a shade-tolerant and N-sensitive plant grown along an N gradient.

## Methods

### Plant materials and growth conditions

Experimental plots were conducted at the teaching and experimental farm of Yunnan Agricultural University in Kunming, Yunnan, China. *P. notoginseng* is a perennial herb; farmers have cultivated this medicinal crop for more than 400 years. One-year-old *P. notoginseng* seedlings were collected from the Wenshan Miao Xiang P*. notoginseng* Industrial Co., Ltd., China (Longitude 104°32 ‘, latitude 23°53 ‘). 1-year-old healthy rootstalks of *P. notoginseng* were selected in our experiments and transplanted to a white plastic pot (30 cm in diameter and 40 cm in depth) on January 2017, and 3 individuals per pot, 120 pots per treatment were arranged.

The soil had the following chemical characteristics: organic mater 0.573%, total N 0.201%, pH (H_2_O) 6.42, total phosphorus (P) 0.727 g/kg, ammonium N 39.93 mg/kg, available potassium (K) 0.019 mg/g, available P 4.88 mg/kg, soil water regime 12%. Pots were placed in environmentally controlled growth permeable black plastic net with growth irradiance of 10% full sunlight. Three N-fertilizer levels were applied in our experiments: (1) LN without N addition, (2) MN with 225 kg·ha^− 1^ N addition in four applications, (3) HN with 450 kg·ha^− 1^ N addition in four applications. N was supplied on April 22, June 22, July 22, August 22, 2017 respectively, along with 225 P_2_O_5_kg· ha^− 1^ (superphosphate) and 450 kg·ha^− 1^ K_2_O (potassium sulphate) in four applications.

*P. notoginseng* grown for 8 months were used to determine plant mortality, leaf morphology and photosynthetic performance, and to collect leaves for comparative transcritome, chlorophyll and elemental N analyses. Five biological replicates were quickly frozen in liquid N and stored at − 80 °C for RNA extraction.

### Leaf anatomy and chloroplast ultrastructure

The juvenile leaves achieved for morphological and anatomical traits were used after 8 months of the N regimes. Leaf anatomical properties were performed in the method of paraffin section, and then the leaves were dehydrated in an alcohol series. Leaf tissues were embedded in paraffin (Thermo Scientific Histostar™) and transversely sectioned at 10 mm thickness by means of microtome (Microm HM325, Walldorf, Germany). Finally, sections were stained with hematoxylin observed under a bright field Microscope (Zeiss Axio Cam HRC, Oberkochen, Germany).

A small piece of 1 ~ 2 mm^2^ was cut between the middle leaf vein and leaf edge and fixed with 2.5% glutaraldehyde and 1% osmic acid. According to the conventional series of ethanol dehydration, epoxy resin embedding, ultra-thin slicer sectioning, sectioning was stained with uranyl acetate and then stained with lead citrate, the chloroplast ultrastructure was observed under JEM100CX-II transmission electron microscope. For the estimation of Sc, 700 nm-thick sections were used by the method of Hanba et al [59].

### Steady-state gas exchange rate

Steady-state gas exchange measurements were carried out using the photosynthesis system (Li-6400-40, Li-Cor, USA) with the 2 cm^2^ fluorescence leaf chamber. The leaf temperature and CO_2_ in the chamber were maintained at 25 °C and 400 μmol mol^− 1^ during measurements, respectively. Subsequently, Photosynthetic light response curves and photosynthetic CO_2_ response curves were performed. Based on photosynthetic light response curves, full induction was complete, an automatic program of light response curves was run to measure the change in gas exchange rate with a set of PPFD. The level of PPFD was listed in the following order: 800, 500, 400, 300, 200, 100, 80, 60, 40, 20 and 0 μmol m^− 2^ s^− 1^, each light intensity stabilized for 5 min. The relationship between *A*_net_ and PPFD was fitted, *A*_net_ = *A*_max_ - *A*_max_C_0_e^-αPPFD/ *A*max^, where *A*_max_ is the maximum net photosynthetic assimilation under saturating light, α is the apparent quantum efficiency (AQY), where AQY was estimated by the slope of the linear region of the light response curve. C_0_ is the index to measure the net photosynthetic rate approaching 0 in low light. According to the parameters in the formula, dark respiration rate (*R*_d_) = *A*_max_- *A*_max_C_0_.

*A*_net_ and *C*_i_ were evaluated at a range of reference CO_2_ concentrations (400, 300, 200, 150, 100, 50, 400, 600, 800, 1000 and 1200 1500 μmol mol^− 1^). CO_2_ response curves and CE were achieved by fitting the data to a nonrectangular hyperbola and the slope the linear region of the CO_2_ response curve, respectively. *V*_cmax_ and *J*_max_ was gained according to the idea offered by Buckley and Diazespejo [106], this calibration requires measurements under low O_2_.

### Chlorophyll fluorescence of PSII

At predawn, minimum and maximum Chl fluorescence yield (*F*_O_ and *F*_m_) was measured in the fully dark-adapted leaves. Minimum, maximum and steady-state fluorescence intensity (*F*_O_`, *F*_V_`, *F*_m_` and *F*_s_) were made in the process of light response curves. *F*_v_`/*F*_m_` was estimated as (*F*_m_` – *F*_O_`)/ *F*_m_`; Φ_PSII_ as (*F*_m_`– *F*_s_)/*F*_m_`; *J*_T_ = PPFD×Φ_PSII_ × α_leaf_ × β, commercial fluorometers usually provide an estimate of PSII total electron transport rate (*J*_T_) by assuming that 400–700 nm (PAR) leaf absorptance (α_leaf_) equals 0.84 [107] and that absorbed photons (β) are equally distributed between the two photosystems (β = 0.5) [108]. This approximation is reasonable for comparison of *J*_T_ between optically similar samples such as leaves of cultivars of a single plant species [109]. Moreover, there was a curvilinear relationship between α_leaf_ and chlorophyll content, whereas the curvature was extremely low when the chlorophyll content was > 0.4 mmol m^− 2^ [30, 110]. According to Evans and Poorter [110], the calculation of α_leaf_ demonstrated that α_leaf_ (0.84, 0.85, and 0.85, in leaves with low, moderate, and high N content, respectively) was similar to the value of 0.84 [111–113]. Therefore, in this study, α_leaf_ also assumed to be 0.84, and β was assumed to be 0.5 [108, 114]. NPQ as (*F*_m_ – *F*_m_`)/*F*_m_`, and qP as (*F*_m_` – *F*_s_)/(*F*_m_` – *F*_0_`). *J*c and *J*o was calculated according to the method of Valentini et al [115], *J*_*O*_ = 2/3 × (*J*_*T*_ − 4 × (*A*_net_ + *R*_d_)), *J*_*C*_ = 1/3 × (*J*_*T*_ + 8 × (*A*_net_ + *R*_d_)). According to the methods of Manter and Kerrigan [116], g_m_ and C_lip_ were calculated as g_m_ = *A*_net_/{*C*_i_-Γ* × [*J*_T_ + 8(*A*_net_ + *R*_d_)]/[*J*_T_-4(*A*_net_ + *R*_d_)]}, C_lip_ = Γ* × [*J*_T_ + 8(*A*_net_ + *R*_d_)]/[*J*_T_-4(*A*_net_ + *R*_d_)], where Γ* is the CO_2_ compensation point. C_c_ was calculated as C_c_ = C_i_ × S*/S. The initial slope of the regression of *J*c/*J*o to C_i_/O was used to S*(Additional file 12: Figure S10), O_2_ concentration (210 mmol CO_2_ mol^− 1^). S was calculated as follow: S=O/2Γ*. g_lip_ can be showed that g_lip_ = C_lip_ × S_c_. g_i_ was calculated by g_i_ = *A*_max_/(C_i_-C_c_) .

### Calculation of N allocation in photosynthetic components

Leaf N was determined with Kjeldahl. SLN was calculated. Photosynthetic-related pigments were determined by the method of Xu et al. [91] and Thayer & Björkman [117]. N_C_, N_B_ and N_L_ were determined according to the method of Niinemets et al [118]. N_photo_ is the sum of N_C_, N_B_ and N_L_. PNUE is the ratio of leaf N used for C fixation per unit leaf area. The formula is as follows:

1 $$ {\mathrm{N}}_{\mathrm{C}}=\left[{V}_{\mathrm{cmax}}/\left(6.25\times {\mathrm{V}}_{\mathrm{cr}}\times \mathrm{SLN}\right)\right]\times \mathrm{SLN} $$

2 $$ {\mathrm{N}}_{\mathrm{B}}=\left[{J}_{\mathrm{max}}/\left(8.06\times {J}_{\mathrm{mc}}\times \mathrm{SLN}\right)\right]\times \mathrm{SLN} $$

3 $$ {\mathrm{N}}_{\mathrm{L}}=\left[\mathrm{Cc}/\left({C}_{\mathrm{B}}\times \mathrm{SLN}\right)\right]\times \mathrm{SLN} $$

4 $$ {\mathrm{N}}_{\mathrm{photo}}={\mathrm{N}}_{\mathrm{C}}+{\mathrm{N}}_{\mathrm{B}}+{\mathrm{N}}_{\mathrm{L}} $$

5 $$ \mathrm{PNUE}={A}_{\mathrm{mPNUE}={A}_{\mathrm{max}}/\mathrm{SLN}\mathrm{ax}}/\mathrm{SLN} $$

V_cr_ is the Rubisco specific activity with a value of 20.8 μmol CO_2_·g^− 1^ Rubisco·s^− 1^. *J*_mc_ is the maximum electron transfer rate per unit cytochrome f (Cyt f) with a value of 155.6 μmol electrons·μmol-1 Cyt f·s^− 1^. Cc is the leaf chlorophyll content (mmol·m-^2^), C_B_ is the combined light system I (PSI), photosystem II (the chlorophyll in PSII) and PSII light-harvesting pigment complex (LHCII) with a value of 2.15 mmol·g^− 1^ N.

### Leaf Rubisco content and activity

The Rubisco content was determined according to Makino et al [119]. Briefly, newly expanded leaves were stored at − 80 °C. 0.5 g frozen leaves were ground in a solution containing 50 mM Tris-HCl (pH =8.0), 5 mM β-mercaptoethanol, and 12.5% glycerol (v/v), and then centrifuged at 1500 g for 15 min at 4 °C. The supernatants were mixed with a solution containing 2% (w/v) SDS, 4% (v/v) β-mercaptoethanol and 10% (v/v) glycerol, boiled in a water bath for 5 min before SDS-PAGE using a 4% (w/v) stacking gel, and a 12.5% (w/v) separating gel. After electrophoresis, the gels were stained with 0.25% Commassie Blue for 12 h, and destained. Gel slices containing the large subunits and small subunits of Rubisco were transferred to a 10 mL cuvette containing 2 ml of formamide and incubated at 50 °C in a water bath for 6 h. The absorbance of the wash solution was measured at 595 nm. Protein concentrations were determined using bovine serum albumin as a standard. Bovine serum albumin (BSA) was measured at 595 nm as standard protein.

Rubisco activity was measured according to Parry et al [120] with minor modifications. The extraction solution contained: 50 mM Tris-HCl (pH = 7.5), 10 mM β-mercaptoethanol, 12.5% (v/v) glycerol, 1 mM EDTA-Na_2_, 10 mM MgCl_2_ and 1% (m/v) PVP-40. Extracts were clarified by centrifugation (8000 g at 4 °C for 10 min) and the supernatant was immediately assayed for Rubisco activity.

### RNA extraction and library construction, sequencing

RNA samples were extracted using RNA pre-pure Plant Kit (Tiangen, Beijing, China). After total RNA was extracted, mRNA was enriched by Oligo (dT) bads, and then the enriched mRNA was fragmented into short fragments using fragmentation buffer and reverse transcripted into cDNA with random primers. Second-strand cDNA were synthesized by DNA polymerase I, RNase H, dNTP and buffer. The cDNA fragments were purified with QiaQuick PCR extraction kit, end repaired, poly(A) added, and ligated to Illumina sequencing adapters. The ligation products were selected by agarose gel electrophoresis, PCR amplified, and sequenced using Illumina HiSeqTM 4000 by Gene Denovo Biotechnology Co. (Guangzhou, China).

### Raw reads filtering and de novo assembly

Low quality reads containing adapters, more than 10% of unknown nucleotides (N), were eliminated. Transcriptome de novo assembly was carried out with short reads assembling program-Trinity. The redundancy was eliminated by the TGICL software and further assembled into a set of non-redundant unigenes.

105G sequencing data were obtained and de novo assembled into 93,162 unigenes (Additional file 13: Table S3) with an average length of 790 bp (Additional file 14: Table S4). Collectively, 41,569 (44.62%) unigenes were functionally annotated in accordance with their parallels with known genes/proteins in the databases. The particular statistics of the functional annotation are emerged as in Additional file 15: Figure S11. After eliminating adaptors, unknown nucleotides and low quality reads, the data generated 43,588,606, 46,978,940, 43,177,242 paired-end 125-bP reads in the LN, MN and HN treatments, respectively, coinciding with approximately 6.48 Gb data (Additional file 16: Table S5)**. Q**20 percentages exceeded 98%, uncalled base (“N”) percentage was equal to 0% per sample (Additional file 16: Table S5). The GC contents were almost identical for all 15 leaves tissues, ranging from 43.08 to 44.20% (Additional file 16: Table S5). In general, between 83.23 and 84.79% of clean reads could be mapped on full gene set (Additional file 17: Table S6). A Pearson’s correlation analysis revealed high correlations between biological replicates (R^2^ = 0.8671 to 0.9769, Additional file 18: Figure S12).

### Basic annotation of unigenes

To annotate the unigenes, we used BLASTx program (http://www.ncbi.nlm.nih.gov/BLAST/) with an E-value threshold of 1e^− 5^ to NCBI non-redundant protein (Nr) database (http://www.ncbi.nlm.nih.gov), the Swiss-Prot protein database (http://www.expasy.ch/sprot), the Kyoto Encyclopedia of Genes and Genomes (KEGG) database (http://www.genome.jp/kegg), and the COG/KOG database (http://www.ncbi.nlm.nih.gov/COG). Protein functional annotations are obtained according to the best alignment results.

### Analysis of DEGs

To identify DEGs within N regimes, the normalized read counts from five replicates of each sample were analyzed and the edge R package (http://www.r-project.org) was used. We identified genes with a fold change ≥2 and a false discovery rate (FDR) < 0.05 in a comparison as significant DEGs. DEGs were then subjected to enrichment analysis of GO functions and KEGG pathways.

### GO enrichment analysis and pathway enrichment analysis

All DEGs were mapped to GO terms in the Gene Ontology database (http://www.geneontology.org), gene numbers were calculated for each term, significantly enriched GO terms in DEGs comparing to genome background were defined by hyper geometric test. KEGG enrichment analysis was carried out through Genomes database (g" http://www.genome.jp/kegg). *P*-value of GO terms and KEGG pathway was gone through FDR Correction, taking FDR ≤ 0.05 as a threshold.

### RT-qPCR assay

To validate the expression of 19 significant DEGs observed in RNA-Seq data, reaction was carried out using EvaGreen 2X qPCR MasterMix Kit (abm, Vancouver, Canada) in a Quanstudio™ 5 Real-Time PCR Intruments (Thermo Fisher Scientific, Inc.). First-strand cDNA was synthesized using the RevertAid™First strand cDNA Synthesis Kit (TransGen Biotech, Beijing, China). DEGs primers were designed using the Primer-Blast (/" https://www.ncbi.nlm.nih.gov/tools/primer-blast/) and synthesized commercially (Shuoqing, Kunming, China). Actin were selected as reference genes [121]. The primers used in qRT-PCR analyses are listed in Table S1. Amplification reaction mixtures were made of 10 μL of Eva Green 2X qPCR Master Mix, 0.5 μL of each forward and reverse primer (10 mM), and 1 μL of cDNA template, and ddH_2_O was added to a final volume of 20 μL. The amplification cycling program was as follows: enzyme activation was operated at 95 °C for 10 mins, moreover, 40 cycles of 95 °C for 15 s, 58 °C for 30 s and 72 °C for 30 s. The results were analyzed using the software accompanying the Quanstudio™ 5 Real-Time PCR instruments. The relative expression values were obtained by using the 2^-ΔΔCt^ method [122].

### Statistical analyses

Statistical analyses were performed with SPSS software package (Chicago, IL, USA) and SigmaPlot 10.0, where the data were tested to confirm their normality and the variables were present as the mean ± SD (*n* = 5–7). We obtained 7 repetiotions that studied physiological parameter for N- cultivated plants, and we generally obtained 5 repetions for bioinformatic analyse. Differences were considered significant when *P* < 0.05 according to the ANOVA F-test. The Ct values derived from qPCR were normalized and the relative fold changes in transcripts were calculated using the relative expression software tool, REST.

## Supplementary information

**Additional file 1: Figure S1.** Leaf phenotypic traits **(a)** and plant mortality **(b)** of *Panax. notoginseng* under nitrogen regimes.

**Additional file 2: Figure S2.** Detection of Rubisco large and small subunits in the leaves of *Panax notoginseng*.

**Additional file 3: Figure S3.** Responses of PSII total electron transport rate (*J*_T_, **a**), rate of electron transport for oxidation reaction (*J*_O_, **b**) and carboxylation reaction (*J*_C_, **c**) to photosynthetic photon flux density (PPFD) in *Panax notoginseng* grown under different levels of nitrogen. Values for each point were means ± SD (*n* = 7). Significant differences are indicated by asterisks (ANOVA; *P* values ≤0.05).

**Additional file 4: Figure S4.** Common differentially expressed genes (DEGs) and their expression profile between moderate- (MN) vs. low- (LN) nitrogen and MN vs. high-nitrogen (HN). Red number indicates up-relation, green number indicates down-relation.

**Additional file 5: Table S1.** KEGG enrichment analysis of the first 13 pathways related to the protective mechanism.

**Additional file 6: Figure S5.** Calvin cycle pathways of *Panax notoginseng* and hierarchical cluster analysis of genes that were differentially expressed under different nitrogen level. Red indicates that the gene has a high expression in the nitrogen level; green indicates that the gene has a lower expression in the nitrogen level.

**Additional file 7: Figure S6.** Differentially expressed genes (DEGs) participating in light reaction under varied nitrogen level. **(a)** MN vs LN and MN vs HN differential gene of photosynthesis pathway for samples of control group, the red box labeled for raising genes, green box labeled as the blue box labeled as there are raised and lowered genes at the same time, the box numbers for the number of the enzyme, suggests that the corresponding gene is associated with the enzyme, and the whole passage is there are many different forms through complex biochemical reactions, an enzyme that differences in genes associated with this pathway are marked by different color box. **(b)** The expression pattern of DEGs involved in photosynthesis pathway. Red indicates that the gene has a high expression in the nitrogen level; green indicates that the gene has a lower expression in the nitrogen level.

**Additional file 8: Figure S7.** The pathway and genes encoding for the photoprotection. In heat map, firebrick indicates that the gene has a high expression in the nitrogen level; navy indicates that the gene has a lower expression in the nitrogen level. **(a)** The expression pattern of DEGs involved in Lutein cycle. **(b)** The expression pattern of DEGs involved in Antioxidant pathway. **(c)** The expression pattern of DEGs involved in Chlorophyll degradation pathway. **(d)** The expression pattern of DEGs involved in nitrate assimilation. **(e)** The expression pattern of DEGs involved in Carotenoid metabolism.

**Additional file 9: Figure S8.** Melt curve of 19 differentially expressed genes (DEGs) and house-keeping gene (Actin).

**Additional file 10: Table S2.** Primers for the RT-qPCR assays of the twenty RNA-Seq libraries used in this study.

**Additional file 11: Figure S9.** Quantitative real-time PCR validation of 19 differentially expressed genes (DEGs) (*n* = 5). Data are mean with bars depicting standard deviation (± SD). Significant differences are indicated by letters (ANOVA; *P* values ≤0.05). The columns represent relative expression obtained by RT-qPCR.

**Additional file 12: Figure S10.** The curvilinear relationships between *J*_c_/*J*_o_ and *C*_i_/O. Every data point represents the mean value of five individual replicates, and small error bars indicate the standard deviation. Initial slopes of **(a)**, **(b)**, and **(c)** represent S* of *Panax notoginseng* grown at low, moderate, and high N concentration, respectively.

**Additional file 13: Table S3.** De novo assembled genes of *P. notoginseng* grown under nitrogen regimes.

**Additional file 14: Table S4.** Assembly quality statics of *P. notoginseng*.

**Additional file 15: Figure S11.** Statistics of the annotation of unigenes in public databases.

**Additional file 16: Table S5.** Summary of sequencing data quality for *P. notoginseng*.

**Additional file 17: Table S6.** Summary ofmapping rate and statistics of expression genes based on the RNA-Seq data.

**Additional file 18: Figure S12.** Pearson’s correlation analysis of the RNA-Seq data.

## Data Availability

All data generated or analyzed during this study are included in this published article and its supplementary information files.

## Abbreviations

A: Antheraxanthin
*A*_max_: Maximum photosynthetic assimilation at the saturating light
At1g32060: Phosphoribulokinase
At1g43670: Fructose-1,6-bisphosphatase
At1g56190: Phosphoglycerate kinase
At3g55800: Sedoheptulose-1,7-bisphosphatase
At4g26520: Fructose-bisphosphate aldolase
ATPase: ATP synthase
CAB: Chlorophyll a/b binding protein
CAB13: Chlorophyll a/b binding protein 13
CAB37: Chlorophyll a/b-binding protein 1
CAB40: Chlorophyll a/b binding protein of LHCII type I precursor
*C*_c_: Chloroplastic CO_2_ concentration
CE: Carboxylation efficiency
*C*_i_: Internal leaf CO_2_ concentrations
C_lip_: Conductance per unit of exposed chloroplast surface area
CPN60A1: Rubisco large subunit-binding protein subunit alpha
DEGs: Differentially expressed genes
FBA: Fructose-bisphosphate aldolase
FBA3: Fructose-bisphosphate aldolase 1
FBP: Fructose-1,6-bisphosphatase
*F*_v_/*F*_m_: Maximum quantum yield of photosystem II
G-6-P: Glucose 6-phosphate
G6PDH: Glucose-6-phosphatede hydrogenase
GAPC: Glyceraldehyde-3-phosphate dehydrogenase
GAPDH: Glyceraldehyde-3-phosphate dehydrogenase
GGPS: Geranyl pyrophosphate synthase
g_i_: Internal conductance
g_i_: Internal CO_2_ condutance
g_lip_: Liquid phase
g_m_: Mesophyll cell conductance
g_s_: Stomatal conductance
GSH: Oxidized glutathione
GSSG: Reduced glutathione
GSTF10: Glutathione S-transferase
GSTU8: Glutathione S-transferase
GTS: Glutathione S-transferase
HN: High nitrogen
IPI: Isopentenyl pyrophosphate isomerase
*J*_C_: Rate of electron transport for oxidation reaction
*J*_max_: Maximum electron transfer rate
*J*_O_: Carboxylation reaction
*J*_T_: PSII total electron transport rate
L: Lutein
LN: Low nitrogen
MN: Moderate nitrogen
N: Nitrogen
N: Neoxanthin
N_B_: N allocation to bioenergetics component
N_C_: N allocation to the carboxylation system
NIA: Nitrate reductase
NIR: Nitrite reductase
NIR1: Nitrite reductase 1
N_L_: N allocation to the light-harvesting system
N_photo_: N allocation to the photosynthetic system
NPQ: Non-photochemical quenching
NYC: Chlorophyll b reductase
Os01g0866400: Fructose-1,6-bisphosphatase;
Os02g0794700: Rubisco accumulation factor 1
PGK: Phosphoglycerate kinase
PGK1: Phosphoglycerate kinase
PPFD: Photosynthetic photon flux density
PNUE: Photosynthetic nitrogen use efficiency
PRK: Phosphoribulokinase
PSY: Phytoene synthase
qP: Photochemical quenching
RAF2: Rubisco accumulation factor 2
RCCR: Red chlorophyll catabolite reductase
*R*_d_: Dark respiration rate
ROS: Reactive oxygen species
RPI: Ribose-5-phosphate isomerase
RPI2: Ribose-5-phosphate isomerase 2
RPI3: Ribose-5-phosphate isomerase 3
Rubisco: Ribulose 1, 5-bisphosphate carboxylase
SBP: Sedoheptulose 1,7-bisphosphatase
S_c_: Chloroplast exposed to intercellular air space per unit leaf area
SLN: Nitrogen content per unit leaf area
TKL-1: Transketolase
TKT: Transketolase
TPI: Triosephosphate isomerase
TPI: Triosephosphate isomerase
TPIP1: Triosephosphate isomerase
V: Violaxanthin
*V*_cmax_: Maximum carboxylation efficienc
VDE1: Violaxanthinde-epoxidase
Z: Zeaxanthin
ZEP: Zeaxanthin epoxidase
*Γ**: Carbon dioxide compensation point
α_leaf_: Leaf absorptance
β: Absorbed photons
*Φ*_PSII_: Effective quantum yield of photosystem II
β-Car: β-Carotene
