# Supplementary figures and images for: Photosynthetic performance and photosynthesis-related gene expression coordinated in a shade-tolerant species Panax notoginseng under nitrogen regimes
Source: BMC Plant Biol. 2020 Jun 28;20:273. doi: 10.1186/s12870-020-02434-z (PMC7321538; doi:10.1186/s12870-020-02434-z)

Additional file 1: Figure S1.

(a)

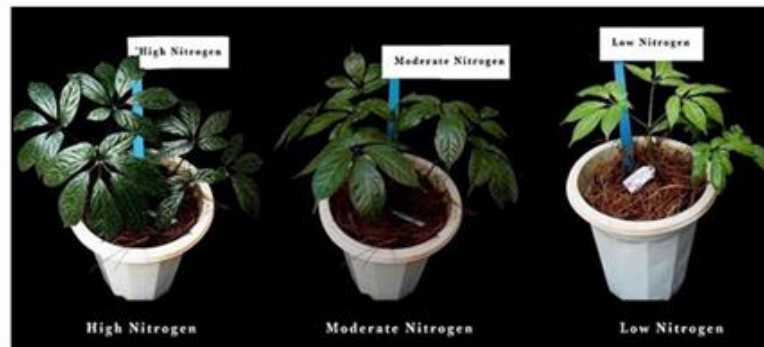

(b)

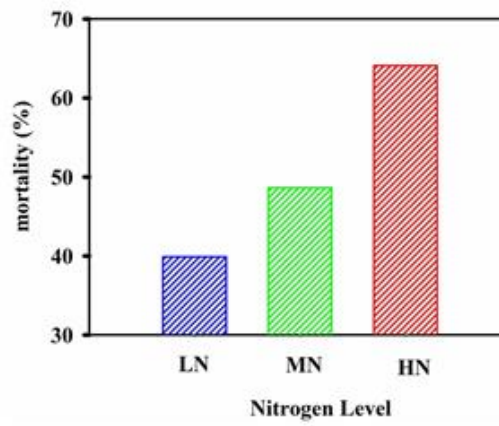

Supplement: Supplementary file 1 — Additional file 1: Figure S1. Leaf phenotypic traits (a) and plant mortality (b) of Panax. notoginseng under nitrogen regimes. [file 12870_2020_2434_MOESM1_ESM.pdf]

**Additional file 2: Figure S2.**

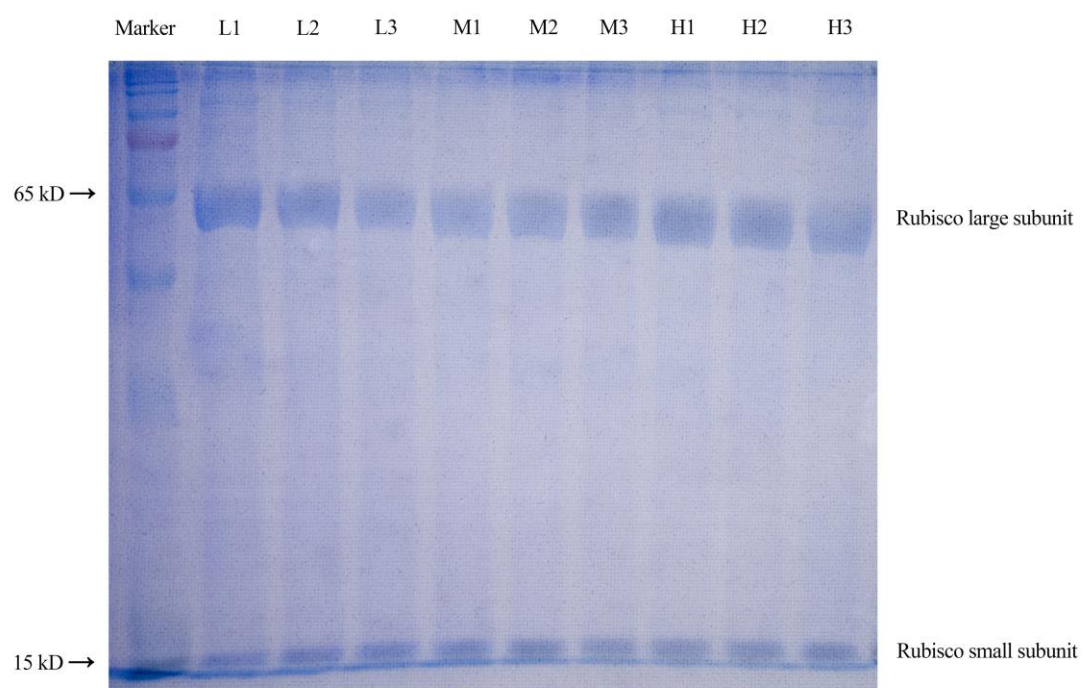

Supplement: Supplementary file 2 — Additional file 2: Figure S2. Detection of Rubisco large and small subunits in the leaves of Panax notoginseng. [file 12870_2020_2434_MOESM2_ESM.pdf]

Additional file 3: Figure S3

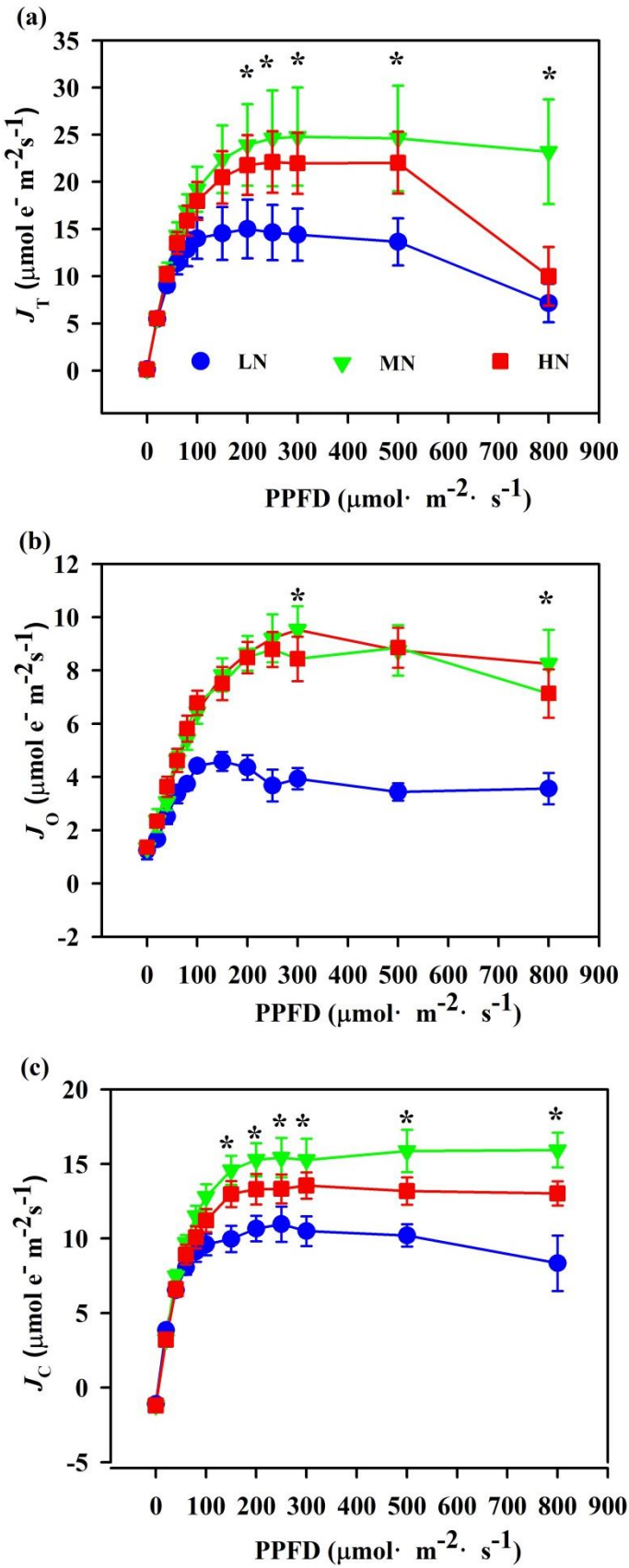

Supplement: Supplementary file 3 — Additional file 3: Figure S3. Responses of PSII total electron transport rate (JT, a), rate of electron transport for oxidation reaction (JO, b) and carboxylation reaction (JC, c) to photosynthetic photon flux density (PPFD) in Panax notoginseng grown under different levels of nitrogen. Values for each point were means ± SD (n = 7). Significant differences are indicated by asterisks (ANOVA; P values ≤0.05). [file 12870_2020_2434_MOESM3_ESM.pdf]

Additional file 4: Figure S4

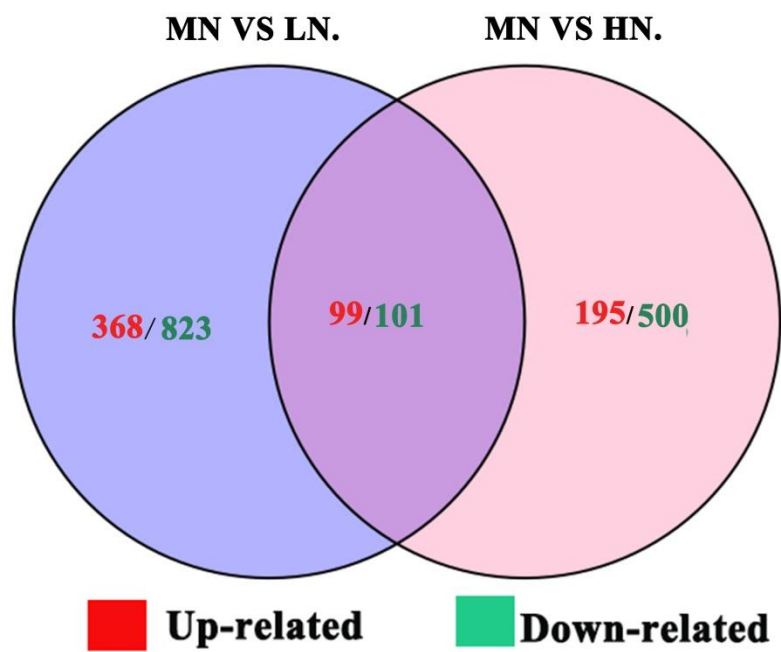

Supplement: Supplementary file 4 — Additional file 4: Figure S4. Common differentially expressed genes (DEGs) and their expression profile between moderate- (MN) vs. low- (LN) nitrogen and MN vs. high-nitrogen (HN). Red number indicates up-relation, green number indicates down-relation. [file 12870_2020_2434_MOESM4_ESM.pdf]

Additional file 6: Figure S5

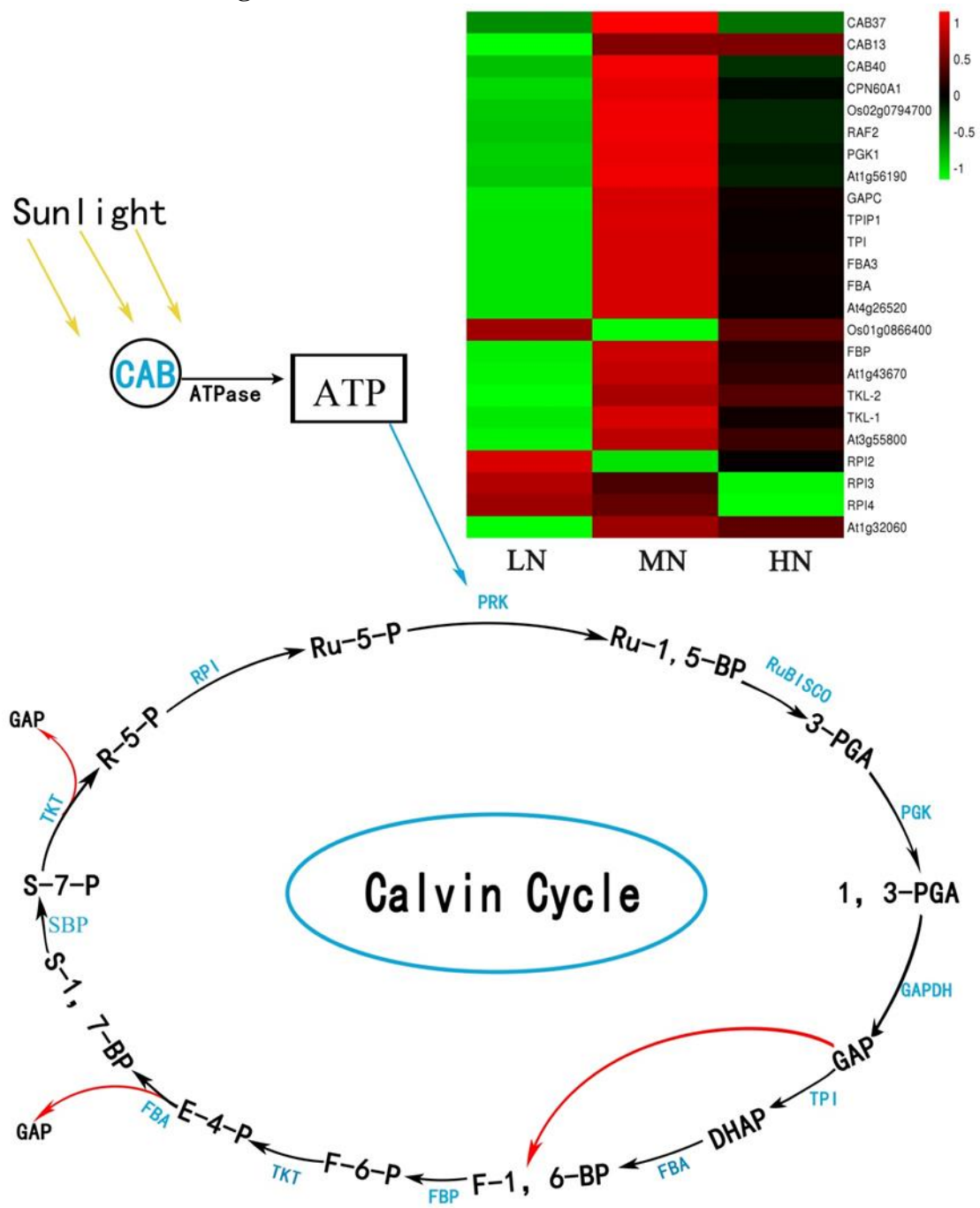

Supplement: Supplementary file 6 — Additional file 6: Figure S5. Calvin cycle pathways of Panax notoginseng and hierarchical cluster analysis of genes that were differentially expressed under different nitrogen level. Red indicates that the gene has a high expression in the nitrogen level; green indicates that the gene has a lower expression in the nitrogen level. [file 12870_2020_2434_MOESM6_ESM.pdf]

**Additional file 7: Figure S6**

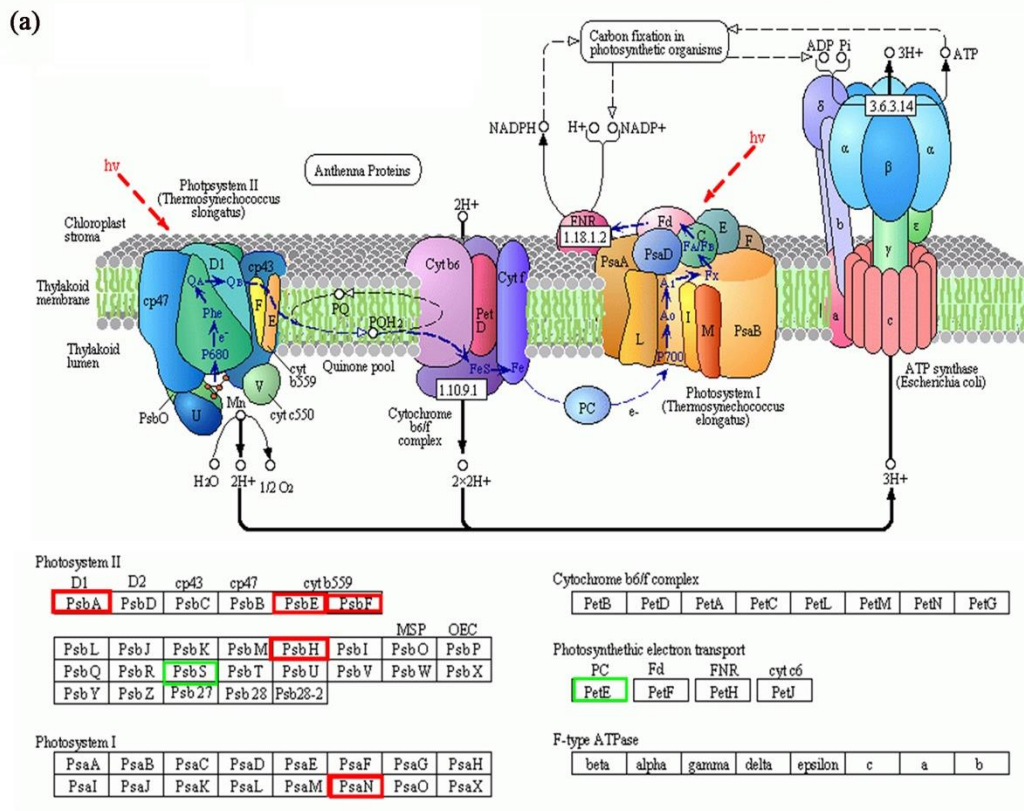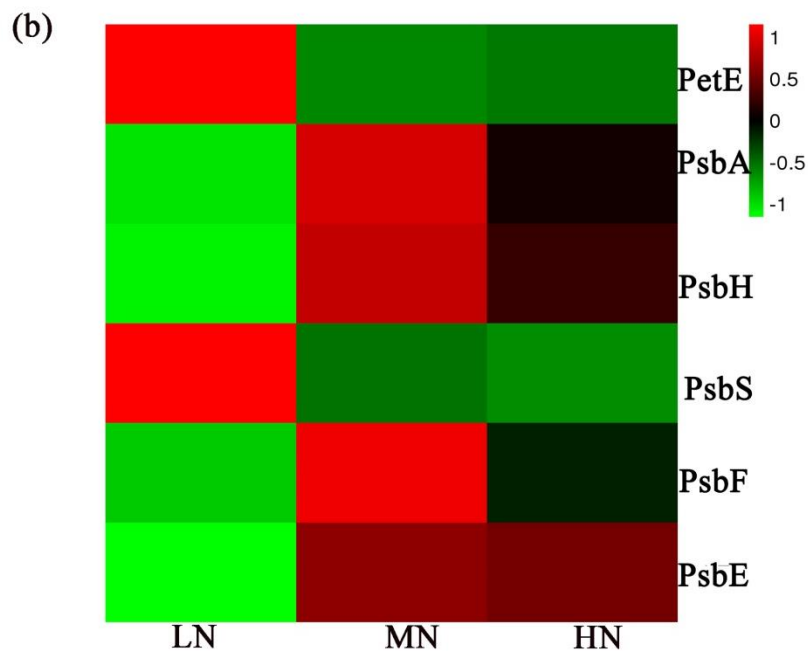

Supplement: Supplementary file 7 — Additional file 7: Figure S6. Differentially expressed genes (DEGs) participating in light reaction under varied nitrogen level. (a) MN vs LN and MN vs HN differential gene of photosynthesis pathway for samples of control group, the red box labeled for raising genes, green box labeled as the blue box labeled as there are raised and lowered genes at the same time, the box numbers for the number of the enzyme, suggests that the corresponding gene is associated with the enzyme, and the whole passage is there are many different forms through complex biochemical reactions, an enzyme that differences in genes associated with this pathway are marked by different color box. (b) The expression pattern of DEGs involved in photosynthesis pathway. Red indicates that the gene has a high expression in the nitrogen level; green indicates that the gene has a lower expression in the nitrogen level. [file 12870_2020_2434_MOESM7_ESM.pdf]

Additional file 8: Figure S7

(a)

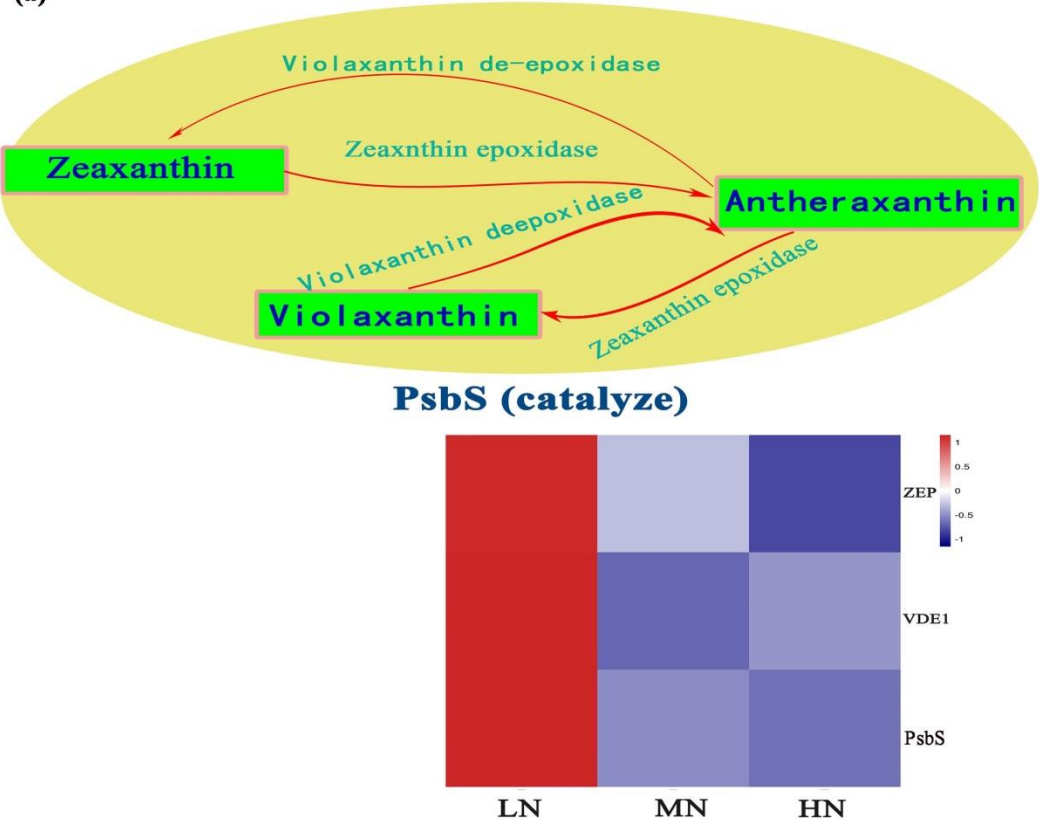

(b)

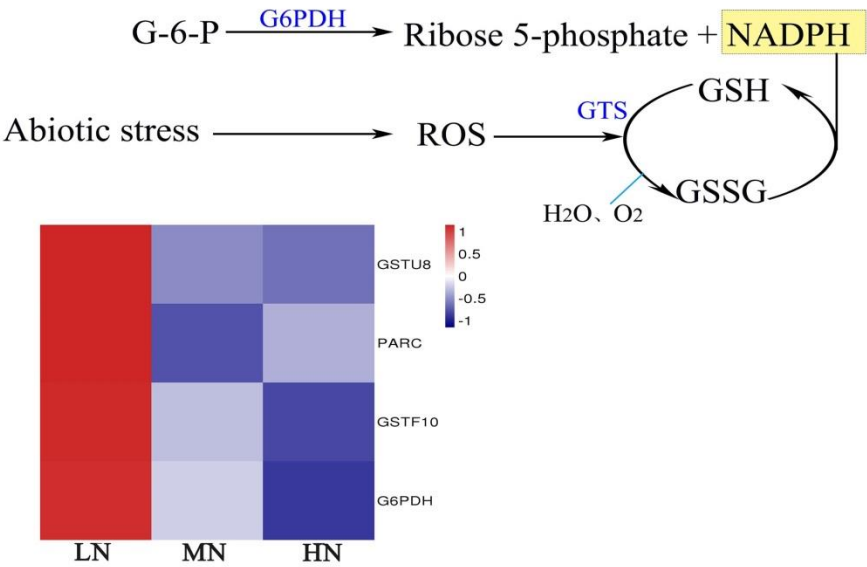

(c)

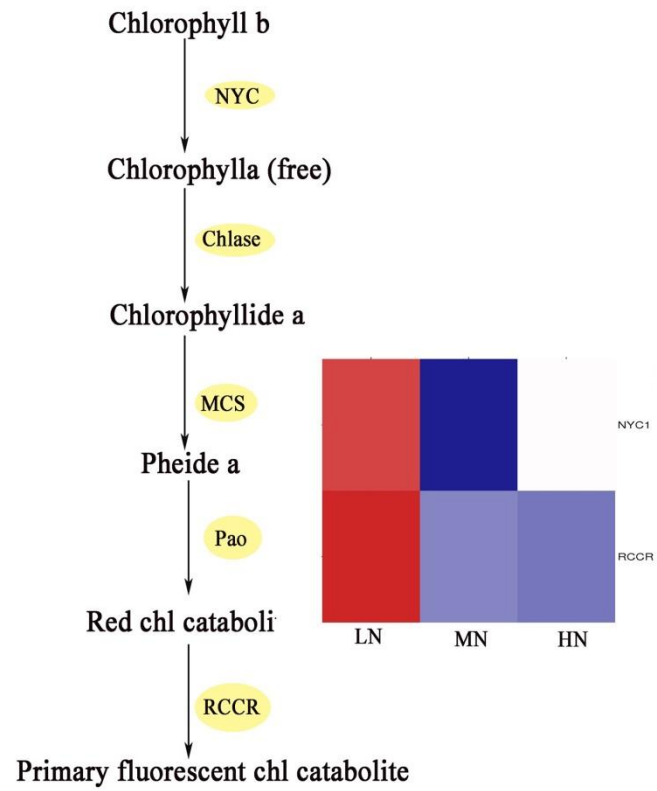

(d)

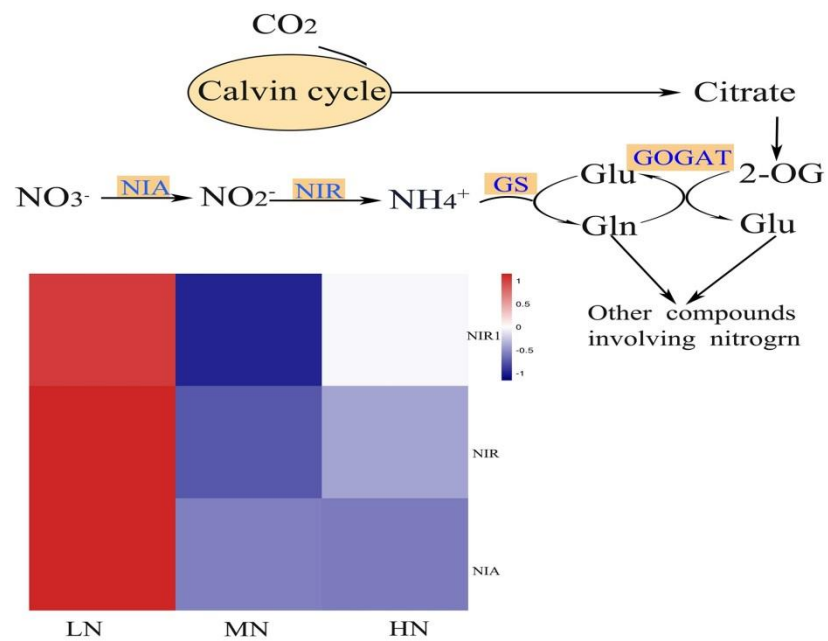

(e)

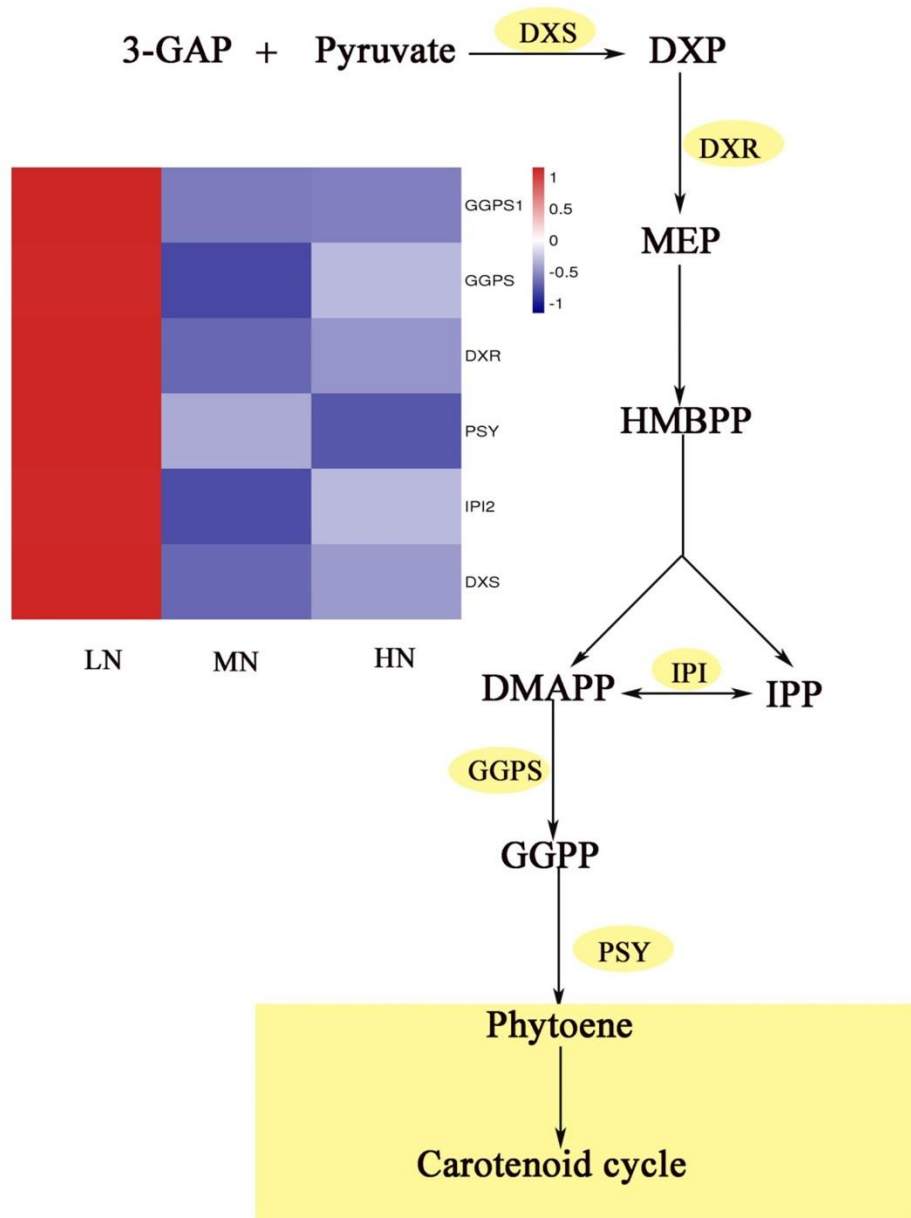

Supplement: Supplementary file 8 — Additional file 8: Figure S7. The pathway and genes encoding for the photoprotection. In heat map, firebrick indicates that the gene has a high expression in the nitrogen level; navy indicates that the gene has a lower expression in the nitrogen level. (a) The expression pattern of DEGs involved in Lutein cycle. (b) The expression pattern of DEGs involved in Antioxidant pathway. (c) The expression pattern of DEGs involved in Chlorophyll degradation pathway. (d) The expression pattern of DEGs involved in nitrate assimilation. (e) The expression pattern of DEGs involved in Carotenoid metabolism. [file 12870_2020_2434_MOESM8_ESM.pdf]

Additional file 9: Figure S8

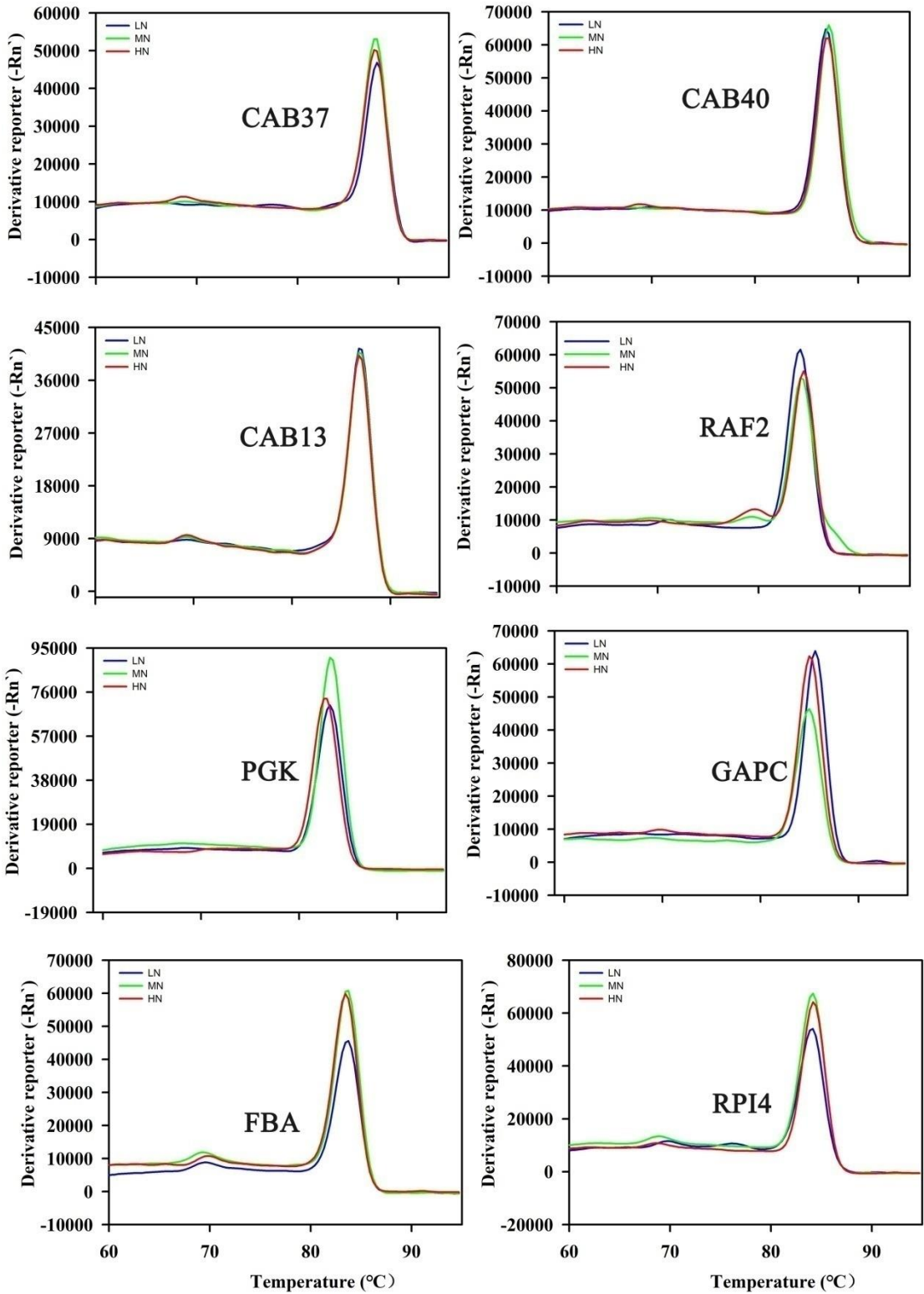

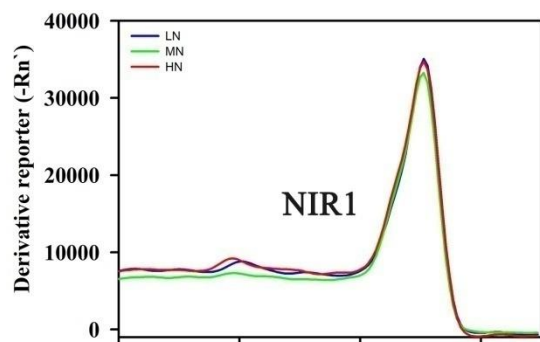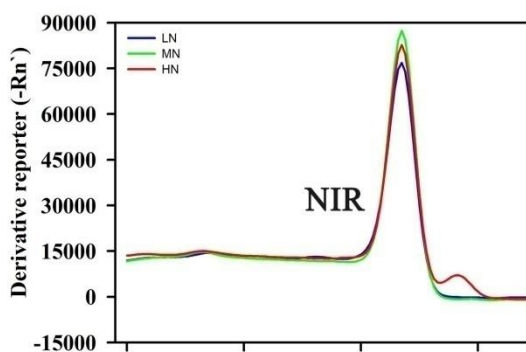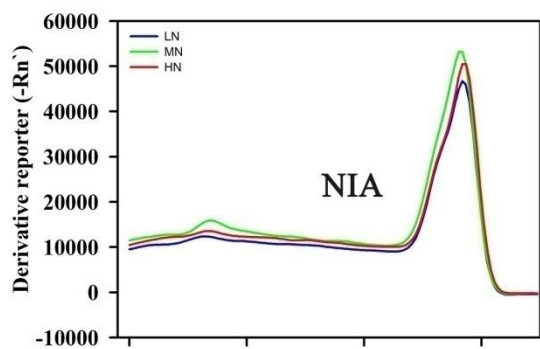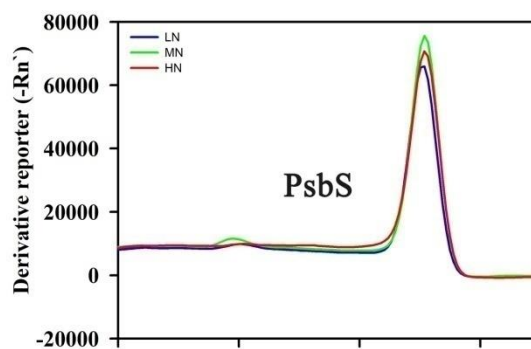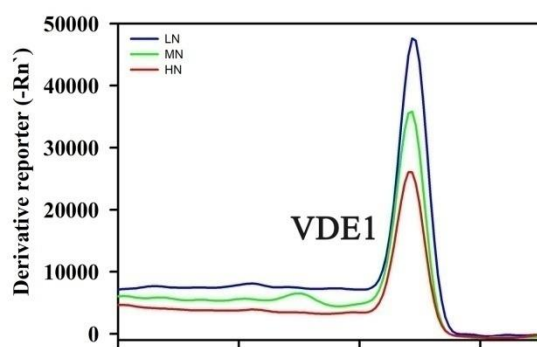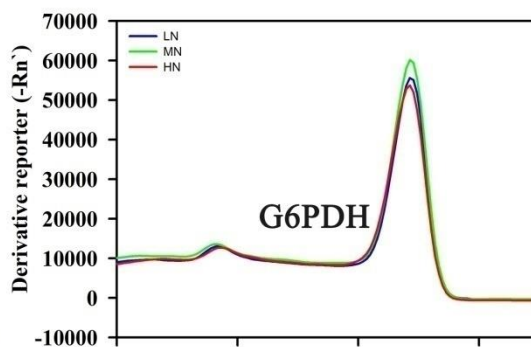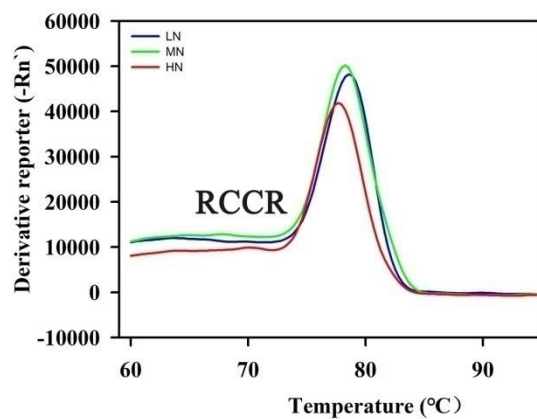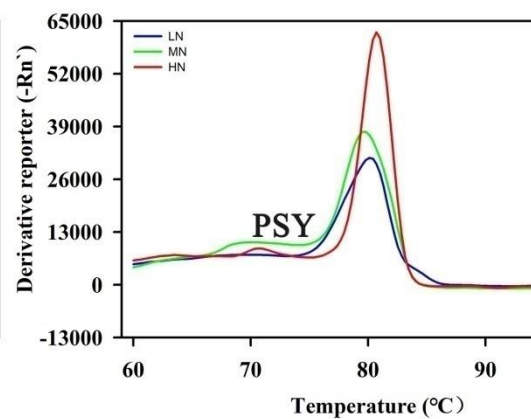

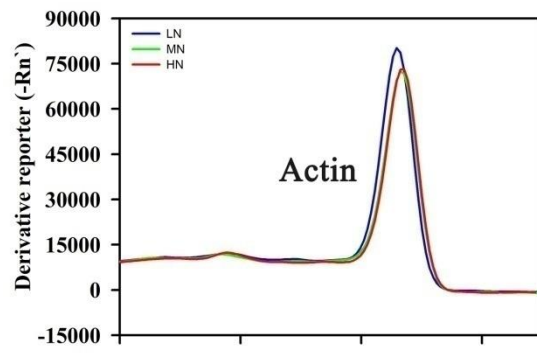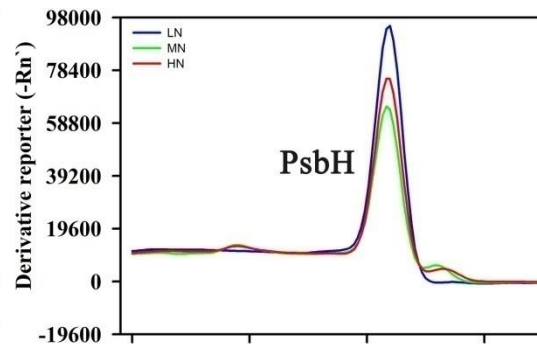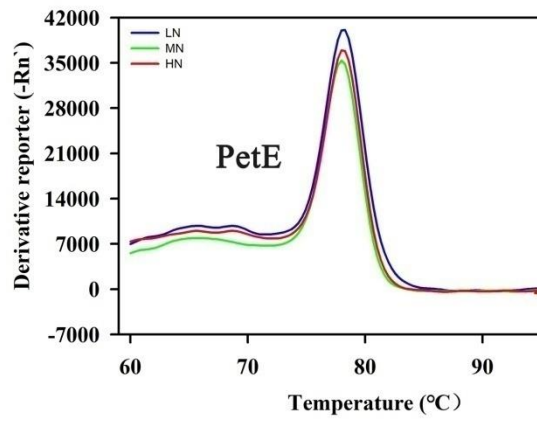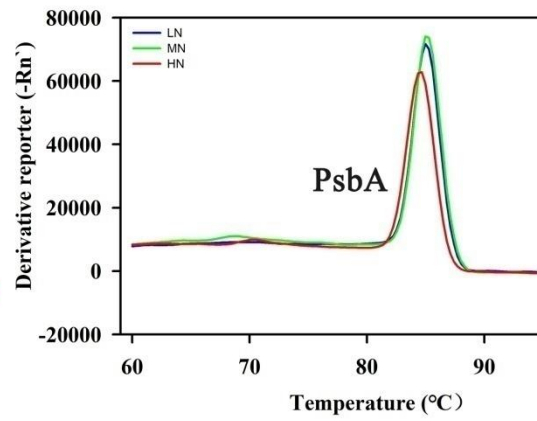

Supplement: Supplementary file 9 — Additional file 9: Figure S8. Melt curve of 19 differentially expressed genes (DEGs) and house-keeping gene (Actin). [file 12870_2020_2434_MOESM9_ESM.pdf]

Additional file 11: Figure S9

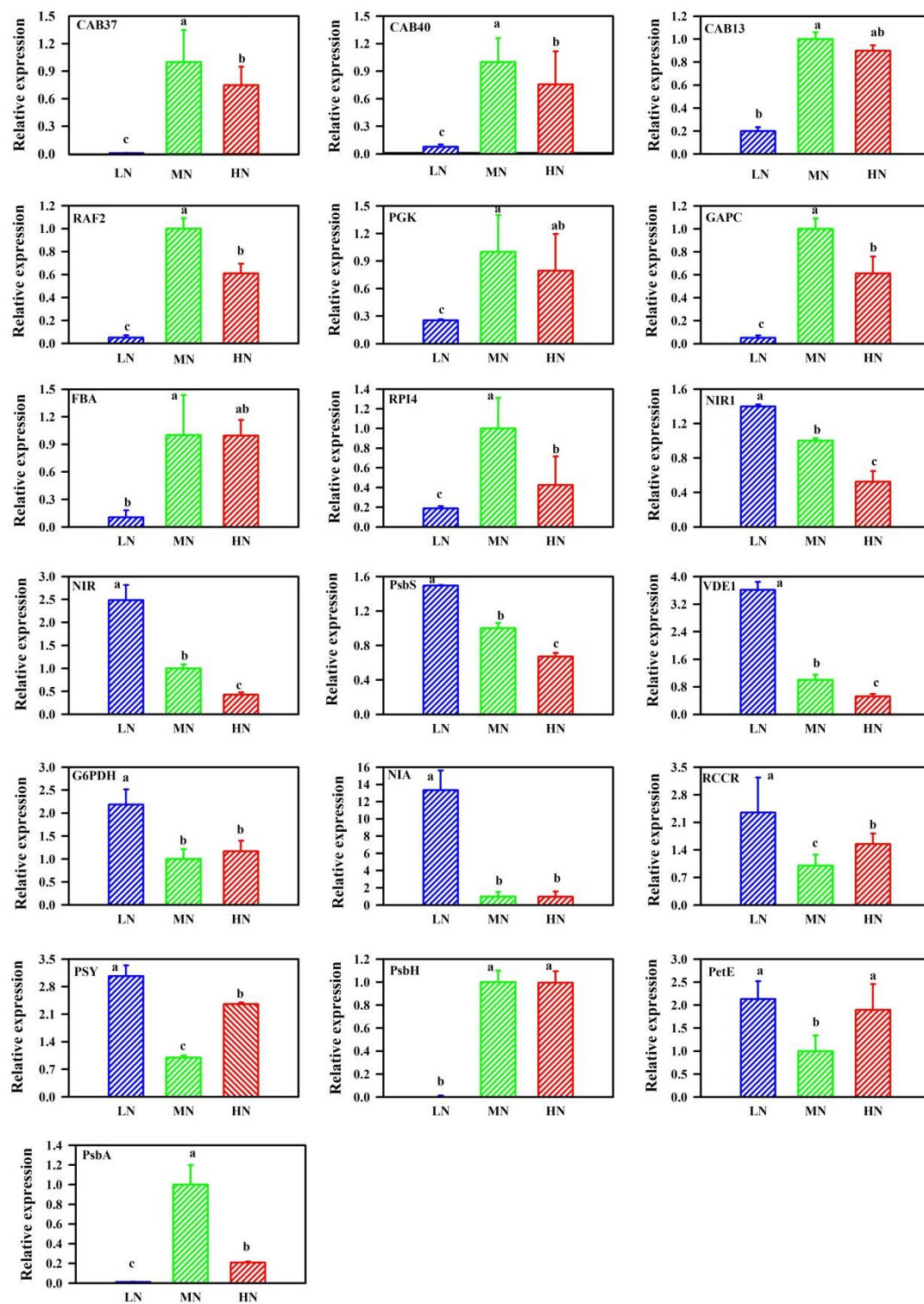

Supplement: Supplementary file 11 — Additional file 11: Figure S9. Quantitative real-time PCR validation of 19 differentially expressed genes (DEGs) (n = 5). Data are mean with bars depicting standard deviation (± SD). Significant differences are indicated by letters (ANOVA; P values ≤0.05). The columns represent relative expression obtained by RT-qPCR. [file 12870_2020_2434_MOESM11_ESM.pdf]

Additional file 12: Figure S10

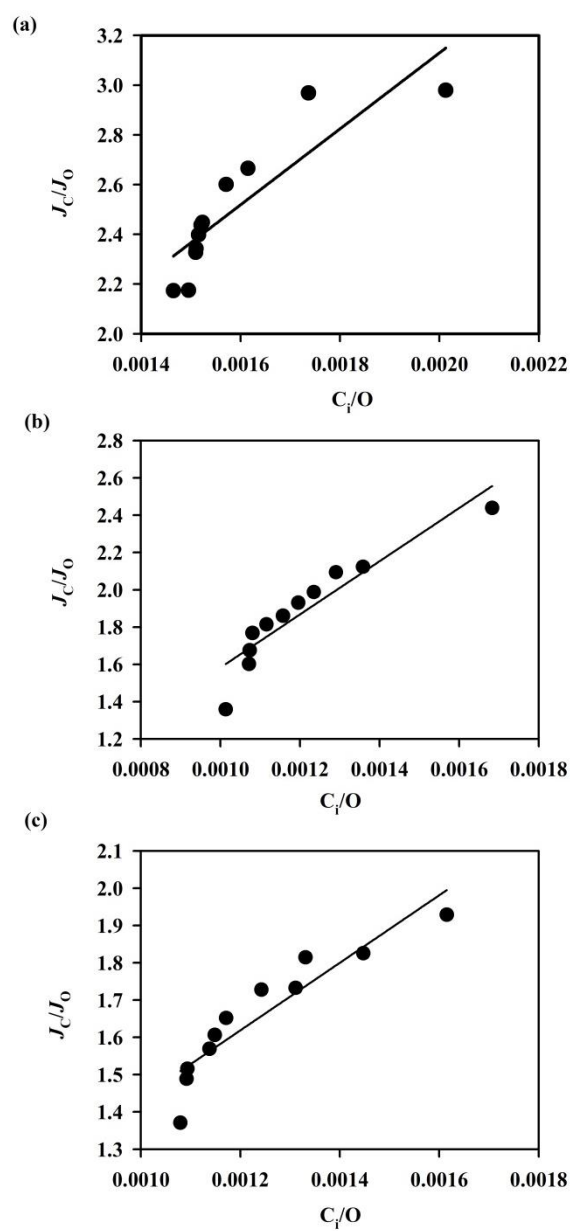

Supplement: Supplementary file 12 — Additional file 12: Figure S10. The curvilinear relationships between Jc/Jo and Ci/O. Every data point represents the mean value of five individual replicates, and small error bars indicate the standard deviation. Initial slopes of (a), (b), and (c) represent S* of Panax notoginseng grown at low, moderate, and high N concentration, respectively. [file 12870_2020_2434_MOESM12_ESM.pdf]

Additional file 15: Figure S11

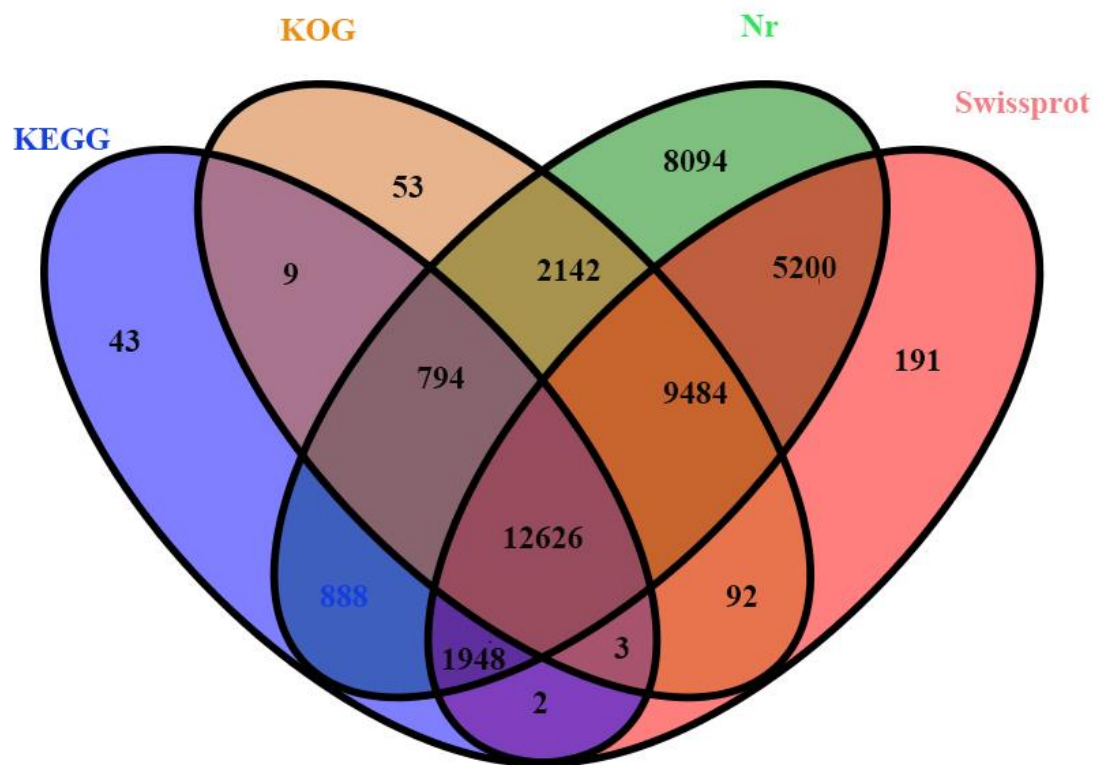

Supplement: Supplementary file 15 — Additional file 15: Figure S11. Statistics of the annotation of unigenes in public databases. [file 12870_2020_2434_MOESM15_ESM.pdf]

## Additional file 18: Fig.S12

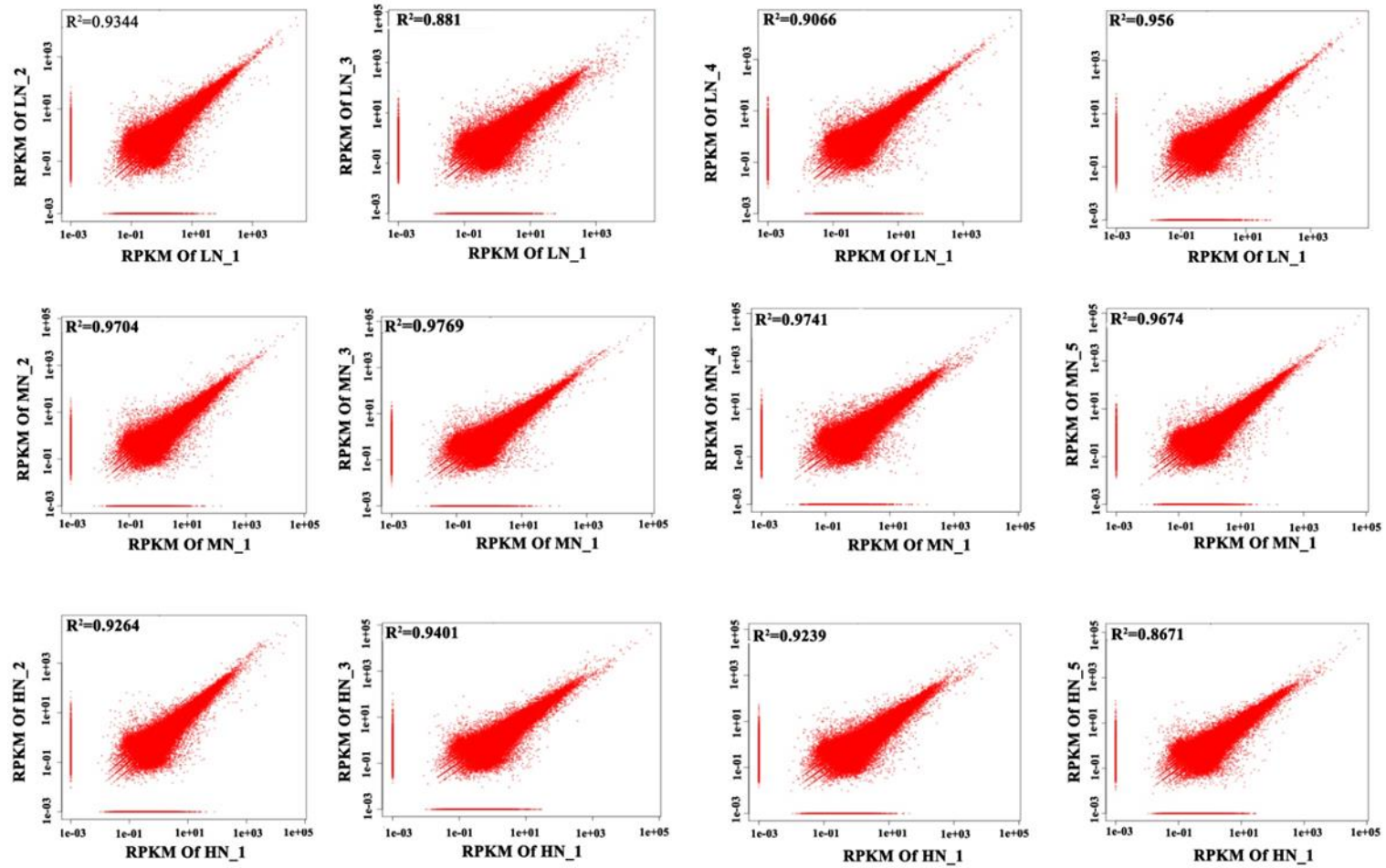

Supplement: Supplementary file 18 — Additional file 18: Figure S12. Pearson’s correlation analysis of the RNA-Seq data. [file 12870_2020_2434_MOESM18_ESM.pdf]
