# Supplementary material for: Photosynthetic performance and photosynthesis-related gene expression coordinated in a shade-tolerant species Panax notoginseng under nitrogen regimes
Source: BMC Plant Biol. 2020 Jun 28;20:273. doi: 10.1186/s12870-020-02434-z (PMC7321538; doi:10.1186/s12870-020-02434-z)
Supplement: Supplementary file 5 — Additional file 5: Table S1. KEGG enrichment analysis of the first 13 pathways related to the protective mechanism. [file 12870_2020_2434_MOESM5_ESM.pdf]

Additional file 5

Table S1. KEGG enrichment analysis of the first 13 pathways related to the protective mechanism.

Pathways	DEGs genes with pathway annotation	Pathway ID
Starch and sucrose metabolism	67 (5.73%)	ko00500
Carbon metabolism	69 (5.9%)	ko01200
Glycolysis/ Gluconeogenesis	37 (3.16%)	ko00010
Photosynthesis	20 (1.54%)	ko00195
Ascorbate and aldarate metabolism	14 (1.2%)	ko00053
Nitrogen metabolism	16 (1.37%)	ko00910
Porphyrin and chlorophyll metabolism	10 (0.85%)	ko00860
Glutathione metabolism	25 (2.14%)	ko00480
Photosynthesis- antenna proteins	2 (0.17%)	ko00196
Carotenoid biosynthesis	12 (1.03%)	ko00906
Pentose phosphate pathway	20 (1.71%)	ko00030
Carbon fixation	16 (1.37%)	ko00710
Oxidative phosphorylation	37 (3.16%)	ko00190
