# Supplementary material for: Photosynthetic performance and photosynthesis-related gene expression coordinated in a shade-tolerant species Panax notoginseng under nitrogen regimes
Source: BMC Plant Biol. 2020 Jun 28;20:273. doi: 10.1186/s12870-020-02434-z (PMC7321538; doi:10.1186/s12870-020-02434-z)
Supplement: Supplementary file 10 — Additional file 10: Table S2. Primers for the RT-qPCR assays of the twenty RNA-Seq libraries used in this study. [file 12870_2020_2434_MOESM10_ESM.pdf]

Additional file 10

Table S2. Primers for the qPCR assays of the twenty RNA-Seq libraries used in this study.

Gene_Name	Forward primer	Reverse primer
Actin	TGCCCGATGGTCAAGTTTATC	GGATTCCTGCAGCTTCCATTC
NIR1	ATGGCTTGCCCACAAAACTG	CTTGGCGTCCATCCACAGAA
CAB37	CCTGGAGACTATGGGTGGGA	AACTTGGCTGGCCCAGATAG
NIR	GGTCTCAATTATGGCTTGCCC	GCAGACGAATACGGATCAGGT
CAB40	CAATGGTGTCAAGTTCGGCG	AGCAAAAGCCTCGGGATCAT
NIA	AACGGTGTTATCCGCACCTT	AACGGATGCTTCCCAGTGAG
CAB13	TGCGAAAGCCCTCAACAAGA	TTCAGCCCAGACTCCATCCT
PsbS	CAGTCCCCGGATTCAATGCT	GGTGATCGTGGTCAGTTCGT
RAF2	AACAACCGGAACCCTCACTC	GGAGCTTGCCCGAGCTATTA
PsbH	AAGGTTGCCCAAAAGCGGTA	GCGACACCCATCAAAGGAGT
PGK1	AACAACCGGAACCCTCACTC	GGAGCTTGCCCGAGCTATTA
PetE	CCCATCATTCACCGGCCTAA	GACGGAACCTCATCCTCGTC
GAPC	CATTGTCGTACCACGCAACC	TGGAATGGCCTTCCGAGTTC
PsbA	GGTCGTGAGTGGGAGCTTAG	AATAGGGAGCCGCCGAATAC
FBA	CACGCAAGCAGTCCACAAAT	TGCCGGATCTTATTCCGACG
RPI4	ACCACCTGCATGAACAACCA	AGATCCACTAGGTGGCGACT
VDE1	GAACTTGGGCCTCGCTCTTA	AGTTGCGTCCTACGCTTTGA
G6PDH	GTTTCTCGCCCCTCCAAAAC	ACATGAAACTGGGACCTTCCT
RCCR	TCCTCGGCCAATGAAGCAAT	GCACCAGTGCTACCGAGATT
PSY	ATAGCTAGCTTGGCGGAACC	GGGGTAAAGGAACCGTGTGT
