# Supplementary material for: Photosynthetic performance and photosynthesis-related gene expression coordinated in a shade-tolerant species Panax notoginseng under nitrogen regimes
Source: BMC Plant Biol. 2020 Jun 28;20:273. doi: 10.1186/s12870-020-02434-z (PMC7321538; doi:10.1186/s12870-020-02434-z)
Supplement: Supplementary file 16 — Additional file 16: Table S5. Summary of sequencing data quality for P. notoginseng. [file 12870_2020_2434_MOESM16_ESM.pdf]

Additional file 16

Table S5. Summary of sequencing data quality for *P. notoginseng*.

Sample Name	Clean Reads	Clean bases	Q20(%)	Q30(%)	N(%)	GC(%)
LN_1	43588606	6538290900	98.73%	96.03%	0%	43.63%
LN_2	52041986	7806297900	98.78%	96.19%	0%	43.47%
LN_3	47055602	7058340300	98.76%	96.13%	0%	43.17%
LN_4	51060152	7659022800	98.76%	96.13%	0%	43.16%
LN_5	45912136	6886820400	98.83%	96.33%	0%	43.44%
MN_1	48516544	7277481600	98.69%	95.96%	0%	43.26%
MN_2	47361480	7104222000	98.73%	96.06%	0%	43.40%
MN_3	51819114	7772867100	98.77%	96.15%	0%	43.85%
MN_4	46905442	7035816300	98.62%	95.80%	0%	43.08%
MN_5	46978940	7046841000	98.73%	96.02%	0%	44.20%
HN_1	45947160	6892074000	98.17%	94.86%	0%	43.37%
HN_2	43177242	6476586300	98.76%	96.10%	0%	44.08%
HN_3	42082018	6312302700	98.80%	96.21%	0%	43.71%
HN_4	47141836	7071275400	98.93%	96.60%	0%	43.76%
HN_5	47196102	7079415300	98.94%	96.64%	0%	43.46%
