# Supplementary material for: Photosynthetic performance and photosynthesis-related gene expression coordinated in a shade-tolerant species Panax notoginseng under nitrogen regimes
Source: BMC Plant Biol. 2020 Jun 28;20:273. doi: 10.1186/s12870-020-02434-z (PMC7321538; doi:10.1186/s12870-020-02434-z)
Supplement: Supplementary file 17 — Additional file 17: Table S6. Summary ofmapping rate and statistics of expression genes based on the RNA-Seq data. [file 12870_2020_2434_MOESM17_ESM.pdf]

Additional file 17

Table S6. Summary of mapping rate and statistics of expression genes based on the RNA-Seq data.

Sample Name	Clean reads	Gene map Rate	Expressed Gene
LN_1	43588606	83.63 %	79632
LN_2	52041986	83.24 %	82045
LN_3	47055602	83.96 %	80177
LN_4	51060152	83.42 %	82250
LN_5	45912136	83.95 %	81191
MN_1	48516544	83.80 %	81092
MN_2	47361480	83.23 %	80918
MN_3	51819114	84.79 %	80830
MN_4	46905442	83.46 %	81421
MN_5	46978940	84.27 %	79002
HN_1	45947160	84.24 %	81719
MN_2	43177242	84.68 %	79258
MN_3	42082018	83.52 %	78776
MN_4	47141836	84.25 %	79996
MN_5	47196102	84.11 %	80496
